# Thermotropic Liquid Crystal-Assisted Chemical and Biological Sensors

**DOI:** 10.3390/ma11010020

**Published:** 2017-12-23

**Authors:** Nicolai Popov, Lawrence W. Honaker, Maia Popova, Nadezhda Usol’tseva, Elizabeth K. Mann, Antal Jákli, Piotr Popov

**Affiliations:** 1 Department of Biology & Chemistry, Ivanovo State University, 153025 Ivanovo, Russia; nikpopov7@gmail.com; 2 Physics and Materials Science Research Unit, University of Luxembourg, L-1511 Luxembourg, Luxembourg; lawrence.honaker@uni.lu; 3 Department of Chemistry & Biochemistry, Miami University, Oxford, OH 45056, USA; popovam@miamioh.edu; 4 Nanomaterials Research Institute, Ivanovo State University, 153025 Ivanovo, Russia; nv_usoltseva@mail.ru; 5 Physics Department, Kent State University, Kent, OH 44242, USA; emann@kent.edu; 6 Liquid Crystal Institute, Kent State University, Kent, OH 44242, USA; ajakli@kent.edu; 7 RavenWindow Inc., Denver, CO 80207, USA

**Keywords:** liquid crystal sensor, optical biosensor, specific sensing, thermotropic LCs, lyotropic LCs, LC sensor numerical simulations, surfactants, antibodies, aptamers, DNA, lipids, chemically functionalized interfaces

## Abstract

In this review article, we analyze recent progress in the application of liquid crystal-assisted advanced functional materials for sensing biological and chemical analytes. Multiple research groups demonstrate substantial interest in liquid crystal (LC) sensing platforms, generating an increasing number of scientific articles. We review trends in implementing LC sensing techniques and identify common problems related to the stability and reliability of the sensing materials as well as to experimental set-ups. Finally, we suggest possible means of bridging scientific findings to viable and attractive LC sensor platforms.

## 1. Introduction

Thermotropic liquid crystals (LCs) are remarkable materials. The discovery of substances in which the optical properties of birefringent crystals are combined with fluid-like behavior have enabled our modern world of information display [1]. Display applications have taken advantage of LCs, beginning with the first twisted nematic display in 1972. Today, liquid crystal displays (LCDs) are ubiquitous in most electronics, from smartphones to large-size television screens. The ubiquity of LC displays arises in part from their responsiveness to external stimuli allied to chemical stability. These valuable LC properties have led to increasing research efforts [2,3,4] into other applications, including chemical and biological sensors.

The unique combination of LC responsiveness to the environment and the striking optical effects that allow the rapid visualization of this response facilitates the use of LCs in sensing applications. The LC responds to several different classes of molecules, including surface-active agents such as lipids and surfactants [5,6,7,8,9] and non-surface-active molecules such as gas vapors [10,11,12,13] and can be tailored to respond only to specific antigens [9,10,14,15,16,17].

**Figure 1:**
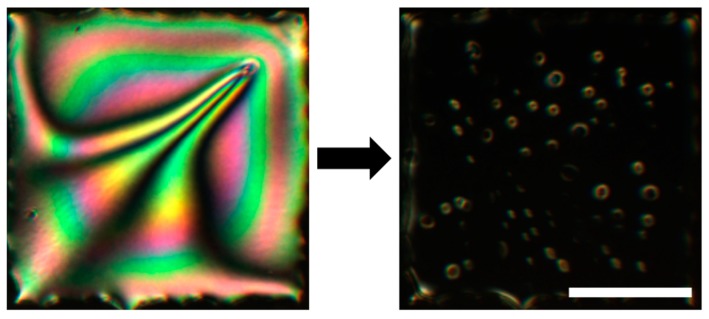
An example of the striking visual change in a 20 µm thick LC film after exposure to an aqueous solution containing a surface-active analyte. See Figure 2 for the details on the basic optical sensing setup and Figure 3 for the schematic representation of the hybrid to homeotropic reorientation of the molecules in the sensing LC film. The microscope images were obtained by placing the sample between crossed linear polarizers. The scale bar represents 200 µm. Adapted from Reference [17].

Several recent books and reviews have summarized and categorized the large variety of approaches to biosensing. Some of these focus on electrochemical, bioelectronic, piezoelectric, cellular and molecular biodetection approaches [18,19,20,21,22,23] and some are devoted specifically to optical biosensors [24,25,26,27,28]. Most approaches fail to meet the full range of required or desirable sensor characteristics. Recently, attention has been directed to liquid crystal-assisted biological and chemical sensors [2,9,29,30,31,32]. As first proposed by Abbott et al. [15], such advanced functional materials hold great promise of overcoming many challenges because LCs readily respond to a variety of chemical and biological agents by reorienting their constituent molecules, which can be easily determined by a beam of light passing through the LC slab during a sensing event. An example of such a transition is shown in Figure 1.

Although we focus on sensor platforms that are based on thermotropic LC materials in this review article, here we also briefly mention another sensing platform that is based on lyotropic LCs. The variety of molecules that are capable of forming lyotropic phases is extremely rich and complex mixtures can be formulated for biomedical applications, drug delivery vehicles and biosensors [33]. Water-soluble discotic shaped molecules may form lyotropic chromonic LC (LCLC) phases [34]. LCLC find applications in biosensing, where larger agglomerates of antibodies, viruses or even cells may need to be identified [35,36]. 

The principle behind this type of sensing is based on visualizing a director defect which is produced when an object in the medium is larger than the extrapolation length b=K/W, where *K* is the director distortion elastic constant in the order of 1–10 pN and *W* is the anchoring strength typically found in the orders of 10^−3^–10^−5^ J·m^−2^. If the size of the microbe before binding is smaller than *b* but becomes larger after clumping with other microbes via binding antibodies, a defect appears that can be detected optically.

Lyotropic-based biosensors were commercialized by Crystal Diagnostics Ltd. (Broomfield, CO, USA) [37] and are currently accredited for the rapid detection of the pathogenic strain of *Escherichia coli (E. coli)* O157 and salmonella in food and for screening of *Listeria monocytogenes* on environmental surfaces.

Progress was made in improvements of the homogeneous and stable alignment on anisotropic surfaces [38]. This is a critical requirement for the applications mentioned above to mitigate the occurrence of false optical signal. Berride et al. reported that addition of small chiral organic molecules—such as amino acids—to achiral disodium chromo glycate (DSCG) phases induces a chiral phase through the formation of tactoids at the interface between isotropic and nematic phases [39]. This interesting effect on the LC director configuration can be used in enantio-selective detection or chirality discrimination.

An interesting lyotropic LC class is realized by the self-assembly of short DNA oligomers [40,41]. It would be also interesting to design a lyotropic LC composed of aptamers and compare the changes in LC textures after introducing antigens that bind to these molecules, with any changes when adding antigens that do not. Such an approach could lead to the development of novel lyotropic aptamer biosensors.

Thus, lyotropic LCs are a very interesting sensor platform with one major advantage: they can be biocompatible, so cells and biological molecules may float within the LC without being destroyed. However, comparatively few studies focus on lyotropic LCs, so the rest of this review is devoted to thermotropic LC sensors.

## 2. Sensor Formats

**Figure 2:**
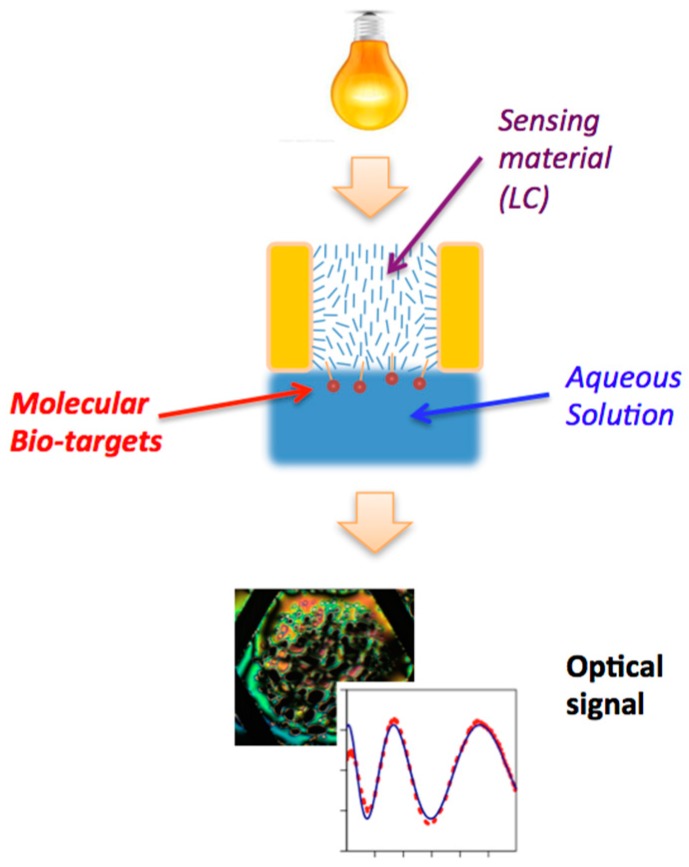
Simplified schematics of an LC-based sensor. The light bulb at the top indicates the microscope light that illuminates the TEM grid filled with LC (middle), in contact with an aqueous solution containing the bio-targets. The light transmitted through the LC is collected either by a microscope objective and recorded by a CCD camera or by a spectrophotometer, leading to the image of an LC texture or of a quantitative optical signal respectively (examples shown at the bottom of the figure.) Note that orientation of the liquid crystal within the cell is determined by the anchoring of the director (average orientation of the LC molecules, indicated by blue dashes within the sample) on the two surfaces as well as by bulk elastic properties. The optical response of the sensor depends on how the director orientation varies within the cell. Adapted from Reference [17].

In most thermotropic LC sensors, the analytes remain outside the LC and instead adsorb on its surface. The molecules that make LCs are very sensitive to surfaces they come in contact with due to the delicate balance between the energies of bulk elastic deformations and surface anchoring of the director [42]. Abbott et al. applied this sensitivity to use LC materials as a transducer element of chemical and biological sensors [7,9,15,17]. LC materials modulate the light that propagates through it due to long-range order in the arrangement of optically anisotropic molecules and, additionally, the LC acts as an amplifier of the interfacial changes; thus, an LC-based sensor provides an optical output signal as schematically shown in Figure 2.

To date, two major thermotropic LC-based sensor formats have been utilized in research experiments: thin LC films in contact with aqueous samples and aqueous LC emulsions. Other designs have been recently proposed, including LCs in micro-capillary tubes [43,44] and fibers [45]. 

Many different methods have been proposed to increase sensor sensitivity, including adjusting the physical properties of the LC and the phases it displays. The nematic phase, with orientational and no positional long-range order, is the most common and, because it usually flows readily, often shows the fastest response. Recently, other LC phases have started attracting interest for specialized modes of sensing. These sensing LC states include, but are not limited to, smectics [17,46,47], cholesterics [48,49,50] and blue phases [51,52].

In this review, we will discuss these different sensing modes. Further, we will discuss some of the limits on analyte properties, along with the influence of solvent and co-solutes. The adaptation of this technique to specific sensing of analytes is critical for its utility; several examples of how this has been done will be given. Finally, the role of numerical simulations in understanding the sensing mechanisms at a molecular level will be highlighted.

### 2.1. Freely-Suspended LC Films

**Figure 3:**
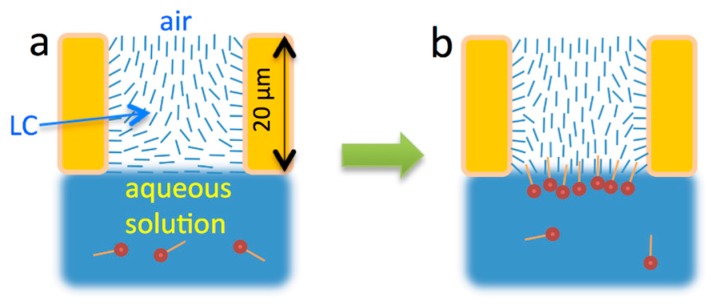
Illustration of nematic LC response to adsorption of amphiphiles. (**a**) LC in hybrid alignment before surface-active molecules bind at the interface; (**b**) LC reoriented due to binding of the surface-active molecules. Adapted from Reference [17].

In the first LC sensor proposed by Abbott et al., LC films were supported by a transmission electron microscopy (TEM) grid and a solid glass substrate coated with an alignment layer [15]. Usually this alignment layer is designed to be homeotropic, aligning the director perpendicular to the glass surface. Later, Hartono et al. proposed freely-suspended LC films supported only by a TEM grid [53,54]. Since both these types of film fill the cells of TEM grids, their thicknesses are approximately equal to the thickness of the grid, which is typically 20 µm (see Figure 3). Both approaches have advantages and disadvantages.

Laboratory preparation of freely-suspended LC films is much less time-consuming than the preparation of the additional glass substrates with alignment layer. As shown in Figure 3, before binding of surfactants to the LC/aqueous solution interface, the alignment of the nematic LC film at that interface is planar and the alignment at the LC/air interface is homeotropic. These initial boundary conditions are well-reproducible across experiments and in widely varying external conditions, such as throughout the whole range of temperatures within the nematic phase of the LC film and for any air humidity levels.

**Figure 4:**
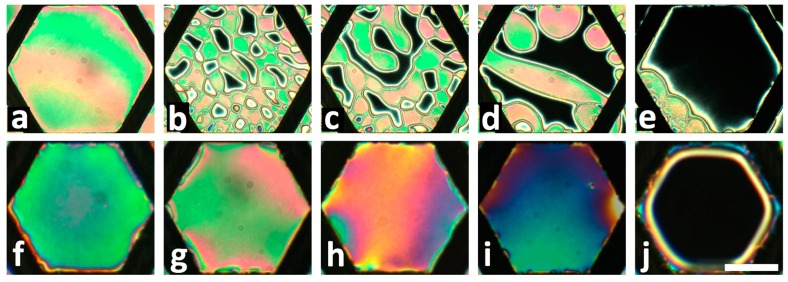
Examples of planar to homeotropic transition of fully submerged freely suspended nematic LC films into an aqueous solution containing surface active analytes. Top row shows a response of an 8CB film to L-DLPC phospholipid at 50 µM at 37 °C after: (**a**) 0 min; (**b**) 4 min; (**c**) 5 min; (**d**) 7 min; (**e**) 10 min. The homeotropic director orientation across the whole film is achieved by the nucleation and growth of homeotropic domains. Bottom row shows the response of a 5CB film to SDS at 22 °C after reaching equilibrium states for the following SDS concentrations: (**f**) 0 mM; (**g**) 0.2 mM; (**h**) 0.4 mM; (**i**) 0.6 mM; (**j**) 0.8 mM. The homeotropic director orientation is established gradually across the whole area of the film without domain formation. All microscope images were obtained by placing the samples between left and right circular polarizers. The scale bar represents 200 µm. Adapted from Reference [17].

Freely-suspended films can be submerged entirely in an aqueous solution but this process must be performed carefully. Since the thermotropic LCs are oily hydrophobic substances, just before submerging a meniscus is formed between the water and the TEM grid that contains the LC films and the water often flushes over the grid as soon as the grid is pushed deep enough. This flushing may displace the LC in the grid causing some of the cells to be overfilled and others to be under filled. This undesired effect may be minimized by submerging the TEM grid in cold water, which upon the initial contact makes LC films stiff: for example, 4-cyano-4′-pentylbiphenyl (5CB) may become a solid or 4-cyano-4′-octylbiphenyl (8CB) may transition to smectic-A. After the water is warmed to room temperature (for experiments using e.g. 5CB as in an example shown in Figure 4f–j or to mammalian body temperature of ~37 °C (for experiments using e.g. 8CB as in an example shown in Figure 4a–e, the LC films return to the nematic phase. Typically, the separate films in cells of the TEM grid appear more uniform in color, indicating better thickness uniformity.

As reported by Popov et al. [55], freely-suspended LC films are not suitable for experiments where the grids are fully immersed in aqueous solutions containing certain surfactants. For example, the addition of the non-ionic oil-soluble emulsifying surfactant Triton X-100 into water did not facilitate the planar-to-homeotropic transition but instead led to 5CB film rupture. One must also be careful with freely-suspended cholesteric LC films, as they may form LC microlenses instead of remaining flat when submerged under water [56].

### 2.2. LC Films with Solid Supports

After the first phase of “trial and error” experiments using freely-suspended LC films, it may be desirable to introduce the solid glass substrate to better support one of the LC surfaces. The solid substrate is typically coated to achieve homeotropic LC alignment, as provided by air but glass substrates can be alternatively treated to provide planar alignment. In some cases, the solid substrate provides not only the alignment layer but are also functionalized with sensing components designed to bind with target analyte, after it diffuses through the bulk of the amplifying LC film and change the director configuration [57].

### 2.3. Grids to Hold LC Films

TEM grids are commonly used for supporting LC films designed for sensing chemical and biological molecules. They are convenient and readily available commercially but they are also limited by their intended purpose in electron microscopy. TEM grid cells are usually square or hexagonal in shape and approximately 20 µm deep. TEM grids are usually made of gold, nickel or copper, which restricts the anchoring energy of LCs with the grid walls. Additionally, copper grids tend to oxidize and degrade quickly when in contact with water. Thus, it may be desirable to prepare custom grids designed specifically to hold the LC in LC-based sensors. For example, Bedolla et al. [58] fabricated chemically patterned micro-wells of precise depths (0.7–30 µm) to hold strained nematic LC films for the sensing of low concentrations of toluene vapors.

### 2.4. LC shells and Droplets 

So far, sensing applications have mainly focused on liquid crystals in flat films, largely due to the convenience of fabrication and visualization of the liquid crystal textures through microscopy. Recent research has explored the use of liquid crystals in curved geometries, such as droplets and shells [59,60]. Monodisperse LC droplets and shells are often achieved with microfluidics chips [46,61]. For sensing applications, they can be used as produced or can be functionalized. Droplets and shells have the advantages of using small amount of fluid, while having a high throughput of consistent product that can be easily tuned just by the adjustment of flow rates. The chips can either be in the form of simple poly(dimethylsiloxane) (PDMS) rubber, normally fabricated through soft lithography processes, or glass capillary-based chips made by techniques developed originally by the Weitz group [62].

Depending on the geometry used, microfluidic chips can be used either for simple droplet production [63,64], for the generation of filaments [64], or for the creation of liquid crystal shells [46,61]. The interface between a liquid crystal shell or droplet and the surrounding medium affects the LC alignment and thus leads to sensing the presence of surface-active molecules, much in the same way as for thin films. For example, Humar et al. [65] used liquid crystal droplets to visualize the adsorption of sodium dodecyl sulfate (SDS) to a 5CB-water interface by observing the anchoring transition from planar to homeotropic/radial. Similarly, Noh et al. [61] visualized the orientation of stabilized 5CB shells as a function of surfactant concentration.

### 2.5. LC in Capillaries and Fibers

In addition to flat and spherical interfaces, cylindrically-shaped liquid crystals, created either by filling capillary tubes or in polymer fibers with LCs, can be also used in sensing platforms.

**Figure 5:**
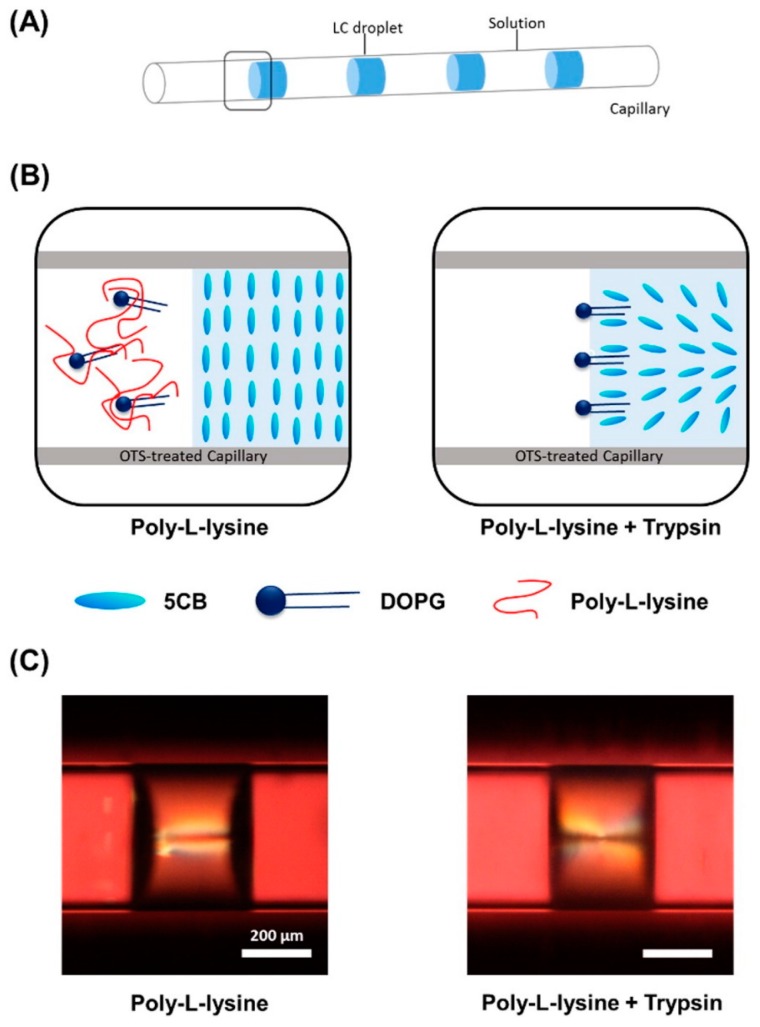
The LC-based capillary sensing system. (**A**) 5CB confined in the micro-capillary; (**B**) schematic illustrations of the ordering change of 5CB droplets confined in an OTS-treated capillary as applied trypsin cleaves peptides on the carboxyl side of lysine; and (**C**) their optical images. Reproduced from [43] with permission from Elsevier.

Due to the capacity of glass to be functionalized to create either planar or homeotropic alignments, a glass capillary can easily be customized to consistently create a desired initial texture before the sensing event. Capillary-based platforms have been used for visualizing the presence of biomolecules. Kim et al. initially presented work on a sensor consisting of a liquid crystal confined in a capillary [43], treated to produce homeotropic radial anchoring, to which surfactant molecules, such as 1,2-dioleoyl-*sn*-glycero-3-phosphoglycerol (DOPG), were adsorbed from a confined solution in the capillary and between LC regions, as shown in Figure 5A. The sensor was able to detect the presence of biomolecules such as trypsin, poly-l-lysine and phospholipids.

**Figure 6:**
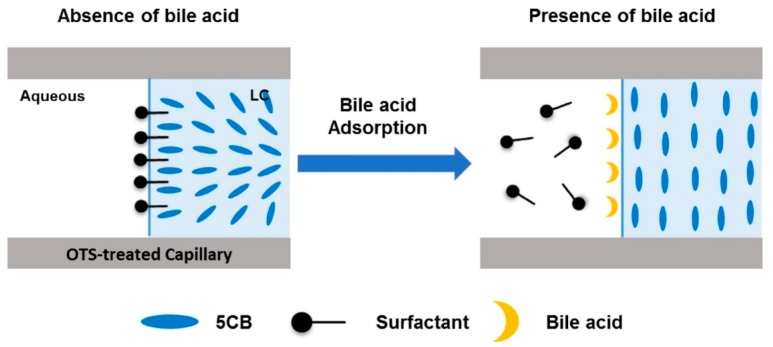
Schematic illustration of the detection of bile acids using the LC-based capillary sensory platform. Reproduced from [44] with permission from Elsevier.

Furthermore, they demonstrated detection of bile acids, such as cholic acid [44]. The presence of the target biomolecule disrupts the homeotropic alignment of the LC caused by alkyl trimethylammonium bromides, such as CTAB, or SDS, displacing the surfactant and restoring a planar texture at the aqueous-LC interface as schematically shown in Figure 6.

Another emerging use of liquid crystals in sensing applications is by encasing the liquid crystal in polymer fibers. The advantages to this approach are clear, as the liquid crystal is stably and securely contained within the polymer, thus creating a portable, robust, easy to use platform.

**Figure 7:**
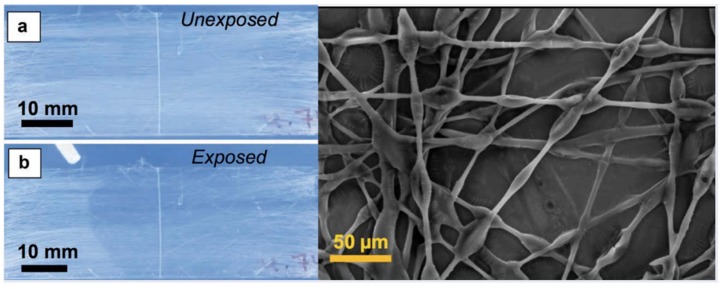
Sensing of toluene vapor with a poly(vinylpyrrolidone) mat filled with 5CB: (**a**) Before toluene vapor exposure, showing a scattering state; (**b**) Upon localized toluene exposure, the area exposed to vapor rapidly transitions from scattering to transparent due to a nematic-isotropic phase transition. When the toluene exposure ceases, the liquid crystal reverts to its scattering state, as in (**a**). The image on the right shows the morphology of the sensing polymer fibers with LC in them. Reproduced from [11] under a Creative Commons License (CC-BY-NC-ND).

One of the first methods for fiber production was through a phase separation process, where the polymer and liquid crystal would be mixed together into a single solution and, as the mixture is spun or sprayed, the liquid crystal would separate from the polymer, remaining in the matrix while still forming distinct liquid crystal droplets [66,67,68]. A one-step process that is relatively easy to perform has allowed the creation of liquid crystal-based textiles for purposes such as temperature sensing, a potentially useful tool in medical diagnostics [67,69]. Alternatively, it has become increasingly popular to produce coaxial fibers, with a liquid crystal core surrounded by a polymer sheath. Extremely thin coaxial fibers can be rapidly and consistently formed through electrospinning techniques, enabling a mobile sensing platform that also produce optical responses visible to the naked eye [11,70,71,72]. Kim et al. [71] and Reyes et al. [11] demonstrated the use of liquid crystals encapsulated in a permeable polymer fiber, such as poly(vinylpyrrolidone), for the sensing of volatile organic compounds (VOCs), as shown in Figure 7.

The response to VOCs was visible even to the naked eye, showing a change from scattering to transparent in the presence of the organic vapor. It was hypothesized that the change in scattering occurs as a result of the mobility of the VOCs through the polymer sheath due to the porosity of the polymer, thus lowering the clearing point of the liquid crystal contained within. Complete transparency is achieved only above 3% of VOC, when the solvent vapor condenses in the fibers and fills the interstitial area with the fluid solvent, thus decreasing the refractive index variation. Removal of the VOCs leads to the system reverting to its initial state over time.

### 2.6. LC-Assisted Direct Visualization of Graphene Features

Graphene is a transparent and flexible conductor that holds promise for various applications, such as in solar cells, batteries, catalysis, hydrolysis, biosensors and bio-imaging [73,74]. Particular interesting applications of LC sensing films are in detecting orientations of graphene and of other similar two-dimensional (2D) sheets and their boundaries [75,76,77]; visualizing flake deformations [78] and identifying their deformation-induced chirality [79,80].

**Figure 8:**
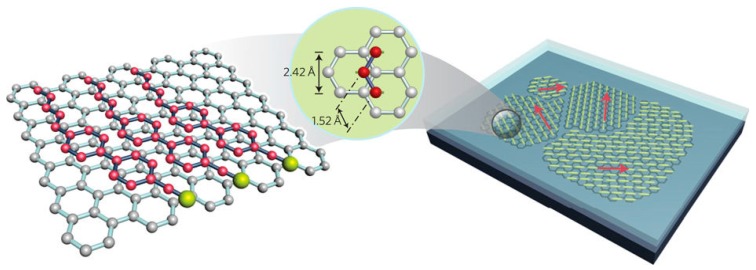
Schematic of liquid crystal alignment on the surface of a graphene layer. The alkyl chains of 5CB occupy alternate positions on the hexagons within the graphene surface. Graphene grown on the surface of a copper foil has multiple domains with different lattice orientations. The alignment directions of liquid-crystal molecules vary in accordance with the graphene domains. Red arrows indicate the aligned director field of the liquid crystal. Short yellow lines indicate liquid-crystal molecules lying (planar anchoring) on a graphene film. Reprinted by permission from Macmillan Publishers Ltd: Nature Nanotechnology [75], copyright 2011.

Kim et al. proposed a simple method for the visualization of arbitrarily large graphene domains by imaging the birefringence of a nematic liquid crystal film that covers the graphene layer [75]. This method relies on a correspondence between the orientation of the liquid crystals and that of the underlying graphene (see Figure 8). Caused by strong π-stacking interaction, the hexagons present in the graphene film might induce highly ordered packing of liquid crystals containing benzene rings and alkyl chains.

Shehzad et al. [77] reported the visualization of grains and boundaries of chemical vapor deposition-grown molybdenum diselenide and tungsten diselenide on silicon using optical birefringence of nematic LC films that cover 2D layers.

**Figure 9:**
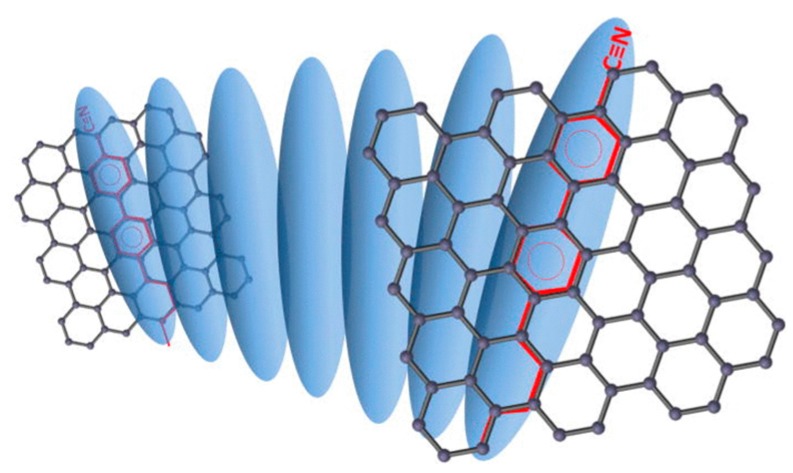
Schematic diagram representing two parallel graphene flakes that induce a helical twist of the LC director due to π-π stacking. The π-π electron stacking is illustrated by matching the benzene rings (red) of 5CB on the graphene-honeycomb structure. Reprinted from Reference [79] with permission of AIP Publishing.

Basu et al. [79] have demonstrated that graphene nano-flakes may serve as chiral dopants for liquid crystals due to strain and edge chirality of the flakes. Thus, nematics and smectics may be used for the detection of graphene deformations and the type of flake boundaries (see Figure 9). The authors also found that LC doped with graphene may have enhanced dielectric anisotropy, faster electro-optic response and enhanced spontaneous polarization [80].

## 3. Increasing Detection Limits and Sensitivity

A great deal of current effort focusses on increasing the detection limits sensitivity. More approaches can be envisioned. The cases considered below have all been tested in the flat-film design but many of them may be useful in other cases, such as droplets or shells, as well.

### 3.1. Flow Cell Design

**Figure 10:**
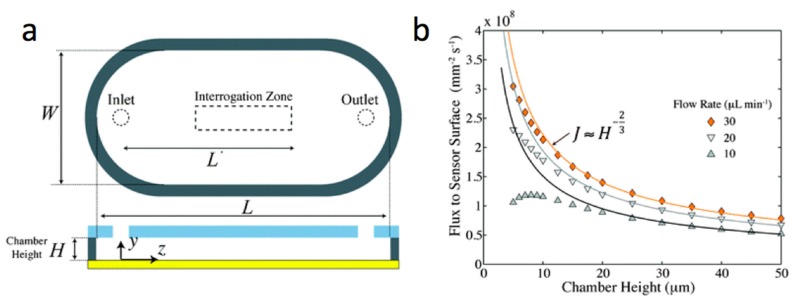
A simple flow-cell design potentially useful for chemical and biological sensors based on thermotropic LC films (**a**) Geometry of the sensing chamber; (**b**) The analyte flux averaged over the interrogation region as a function of the chamber height H. The interrogation region may include the LC sensor. Adapted from Reference [82] with permission of The Royal Society of Chemistry.

Currently, most approaches to thermotropic LC-based sensing rely on the diffusion of target analytes through the bulk of a sub-phase to the aqueous/LC interface. Early research on surface plasmon resonance (SPR) biosensors faced similar limitations but as the interfacial phenomena became better understood, attention turned to designing micro-arrays [81] and flow-cells [82,83] to further increase the limits of detection (LOD) and decrease the amount of samples required for experiments. The work of Lynn et al. proposes a simple flow-cell design which could also be used in chemical and biological sensors based on thermotropic LC films [82]. Their experiments demonstrated (see Figure 10) that LOD scales as *J ≈ H*^−2/3^, where *H* is the height of the flow-cell and *J* is the flux of short ssDNA oligomers to the sensor surface. Such findings may be very useful in the development of optimized flow-cells for LC-based sensors. However, the flow cells must be carefully designed since sufficiently high flow rates may align the LC film [84]. The flow alignment is characterized by the Ericksen number, which quantifies the competition between the flow-induced and boundary-induced LC orientation. Flow-cell chambers could be extremely useful to pass a sequence of different aqueous samples through the chamber for a predetermined self-assembly of biological recognition moieties on the aqueous/LC interface [14].

### 3.2. Combining LC Films with Other Techniques

Another approach to increasing sensor sensitivity involves combining two or more different techniques. For example, the study by Abuabed explores the performance of an LC-assisted SPR biosensor [85]. In this work, the nematic LC is used as a dielectric layer coating on the gold surface functionalized with receptors for specific sensing. The evanescent wave in the LC experiences a refractive index that changes due to LC molecular realignment. Thus, in the case of presence of biological or chemical agents, the SPR signal depends not only on the binding events of analytes to receptors but also on the changing refractive index due to the loss of LC alignment, which combined may lead to amplified SPR sensitivity.

### 3.3. Choice of Sensing LC Materials

The molecule 4-cyano-4′-pentylbiphenyl (5CB) is the thermotropic LC most commonly used in research and development of LC-based biosensors. It has served well to prove the general concept. Developing a practically useful LC sensor of chemical or biological molecules may, however, require adjusting the LC sensing material.

#### 3.3.1. Tuning LC Elastic Constants

**Figure 11:**
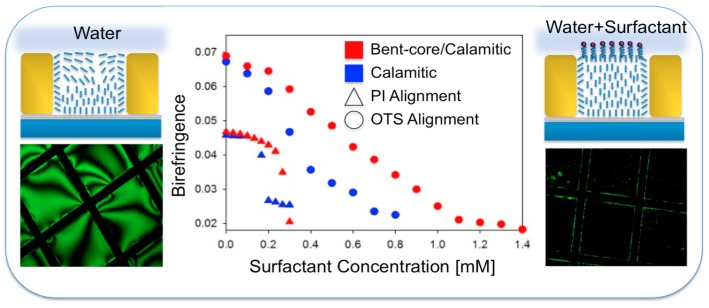
Improving LC-based biosensing in aqueous phases by adding bent-core materials to the sensing LC film. Reprinted with permission from Reference [6]. Copyright 2012 American Chemical Society.

Iglesias et al. [6] studied the effect of adjusting the elastic constants of the LC films by adding bent-core LC to 5CB. They found that the range of sensitivity to a simple surfactant, SDS, increased and a nearly linear response signal was achieved as shown in Figure 11.

#### 3.3.2. Dichroic LC and Polarizers

Typically, polarizers are required to view the LC textures that form due to certain interaction of the target analyte with the sensing LC interface. We have shown that circular polarizers may be preferred over the linear polarizers in cases when the azimuthal director orientation of the LC is not important [5]. In such case the intensity of the optical signal becomes higher and the measurements conducted with a spectrophotometer become more accurate [47].

Wu et al. showed that doping the LC with a dichroic dye [86] allows the user to remove both polarizers completely and thus further increase the transmittance of light before the binding of the bovine serum albumin (BSA) takes place.

#### 3.3.3. LC Temperature and Phase

**Figure 12:**
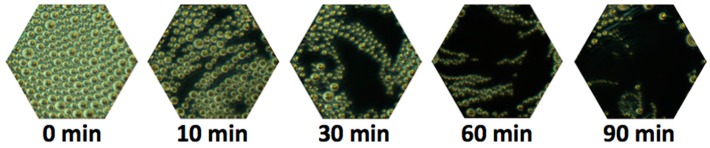
TFCD coverage as a function of time with 100 nM of the phospholipid DLPC at 37 °C. Smectic-A LC film as viewed using circular polarizers. The diameter of an inscribed circle in the hexagons is 100 µm. Adapted from Reference [17].

5CB is a nematic LC in the range of room temperatures but many biological molecules function best at physiological temperatures, typically ~37 °C. An obvious replacement for 5CB would thus be 8CB (4-cyano-4′-octylbiphenyl), which shows a nematic state at the desired range of temperatures [17]. In fact, at room temperature, where 8CB is in the smectic A phase, this LC senses surfactants at concentrations well below the sensitivity of the typical nematic state [9,47]. This is possible because tiny amounts of surface-active agents at the LC/water interface suppress toric focal conic domain (TFCD) formation in hybrid-aligned 8CB films (see Figure 12).

**Figure 13:**
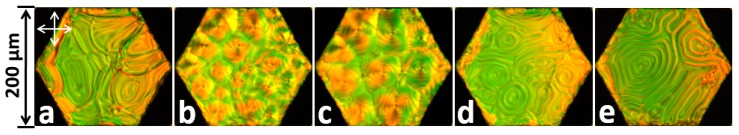
Texture change of a thin film made with 5CB with a chiral dopant KDI-6-A. SDS concentrations in distilled water: (**a**) 0 mM; (**b**) 0.32 mM; (**c**) 0.63 mM; (**d**) 1.18 mM; (**e**) 1.89 mM. The arrows represent the crossed orientations of polarizers. Reproduced from Reference [55] with permission of the Nanomaterials Research Institute of Ivanovo State University, Russia.

Nematic LCs, such as 5CB, can be turned into chiral (N* or cholesteric LCs) when mixed with chiral dopants [4,48,87]. The response of cholesteric films in contact with aqueous solutions of surfactants is rather peculiar (See Figure 13). Here, the presence of SDS is signaled not via hybrid-to-homeotropic realignment but by the change in the pitch of the periodic “fingerprint” texture. This platform might be able to distinguish the sense of chirality of biological molecules, which are often chiral themselves. We note that cholesteric LC films submerged under water may not remain flat and instead form LC microlenses (µ-lenses) [56] with focal length depending on the chirality, which may also be used for chirality sensing.

In another study by Tran et al. of cholesteric LC shells [60], the effect of induced anchoring transitions at the inner and outer boundaries is controlled by changing the surfactant concentration or by raising the temperature close to the clearing point. The authors use the Landau-de Gennes model of cholesterics to simulate and explain the various surface structures that are produced due to the shell confinement and anchoring conditions.

Cholesteric LCs may provide relatively inexpensive, efficient and highly sensitive gas or environmental sensors [4,12,87,88] based on the ability of cholesteric phases to selectively reflect polarized light. An important application could be to detect the presence of specific volatile organic compounds (VOC) in the human body that can serve as a biomarker for certain diseases, such as lung cancer and diabetes [89,90,91,92].

The detection principle is based on the slight changes in LC orientation upon interaction with diffusing gas molecules, resulting in the change of the peak wavelength of selective reflection (λ_max_) of cholesteric LCs. The changes in the reflection band of the chiral nematic phase are attributed to the alterations of the length of the helical pitch of the LC phase under the influence of external stimuli [88]. The shrinkage of the cholesteric pitch leads to a spectral blue-shift compared to the original central reflection wavelength, whereas a slight loss of LC molecular order results in a red-shift [88,93,94].

This principle was adopted in the work by Kek et al. [95,96], using carefully-chosen cholesteric LCs to detect a wide variety of VOCs. It was found that the reduction of the elastic constant of cholesteric LCs resulted in a red shift of the λ_max_, whereas a blue shift of λ_max_ was observed upon contact of VOCs with cholesteric LC mixtures that consist of Schiff-base, ester linkages, tolan or phenylcyclohexyl derivatives [95]. The authors made efforts to improve the sensitivity of the VOC detection by adding phenylpyrimidine to the cholesteric LCs. Due to the reduction of the nematic-isotropic transition temperature, distinct changes in peak wavelength were observed [95]. This provides evidence that the method described above has the potential to provide selective and sensitive means of VOC detection.

Lee and coworkers explored biosensing by blue phases (BPs), which are another LC phase produced by adding chiral dopant to a nematic LC formulation [51]. The BPLC-based biosensing approach was developed for the detection of bovine serum albumin (BSA). The researchers have found that the reflected color of the BP is blue-shifted in presence of BSA.

#### 3.3.4. Tailored LC Mesogens

**Figure 14:**
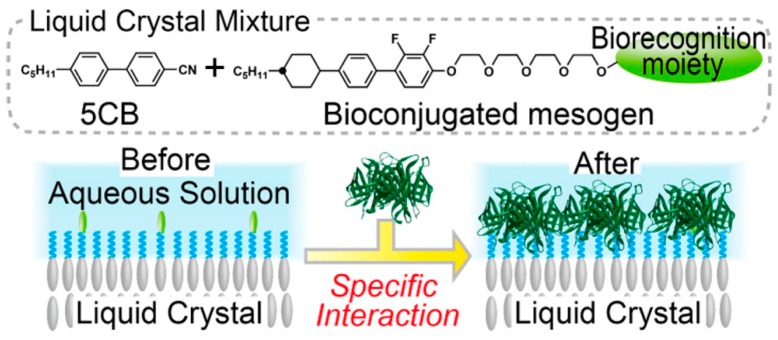
Bioconjugation of amphiphilic mesogens with specific binding moieties. Reprinted with permission from [97]. Copyright 2012 American Chemical Society.

Eimura et al. proposed bio-conjugation of LC molecules [97] to demonstrate the basis of a general and facile method for the introduction of biological recognition functionality at the aqueous-LC interface (See Figure 14). Munir et al. managed to decorate the 5CB microdroplets with 3-aminophenyl boronic acid (APBA) for glucose detection with high specificity and sensitivity [98]. Further optimizations of synthetically tailored amphiphilic mesogens and their compositions are required to fully control the adsorption of biomolecules at aqueous-LC interfaces and to generate alignment transitions in the LCs triggered by protein, sugar or other biological molecule binding events.

#### 3.3.5. Ionic Liquid Crystals

Recently there has been a growing interest in ionic liquid crystals (ILCs) [99] and their applications in chemical and biological sensing [100,101,102]. ILCs are LCs composed of salts of cations and their counter ions. The ionic nature of such materials adds useful properties such as low volatility, high thermal and chemical stability, a wide electrochemical window, etc. The properties of the ILCs can be tuned by choosing different cationic and anionic moieties.

Munje et al. [102] described a sweat-based diagnostic biosensor using room temperature ILCs (RTILCs). It was shown that these compounds may provide prolonged stability in detection of analytes, such as interleukin-6 and cortisol, at varying sweat sample pH levels. The RTILCs can be designed to have a high biochemical compatibility with enzymes and proteins for improved operational integrity of such capture probes as antibodies.

Atta et al. [101] fabricated an electrochemical sensor with a carbon paste electrode modified with ILC (2-chloro-1,3-dimehyl-imidazolidinium hexafluorophosphate) for the selective determination of Terazosin (TZ) a drug used to treat high blood pressure. The use of this ILC increases the sensitivity and selectivity of the electrode to TZ in the interfering presence of SDS. This is achieved by the ability of the chosen ILC to increase the electro-catalytic activity of the electrode surface and create an excess accumulation of TZ in the vicinity of the probe.

## 4. Analytes and Solvents 

The development of LC-based biosensors requires close attention to the type of the surface-active analytes and to their solvents. Both may influence the sensitivity and even the integrity of the LC biosensor.

### 4.1. Hydrophilic-Lipophilic Balance

**Figure 15:**
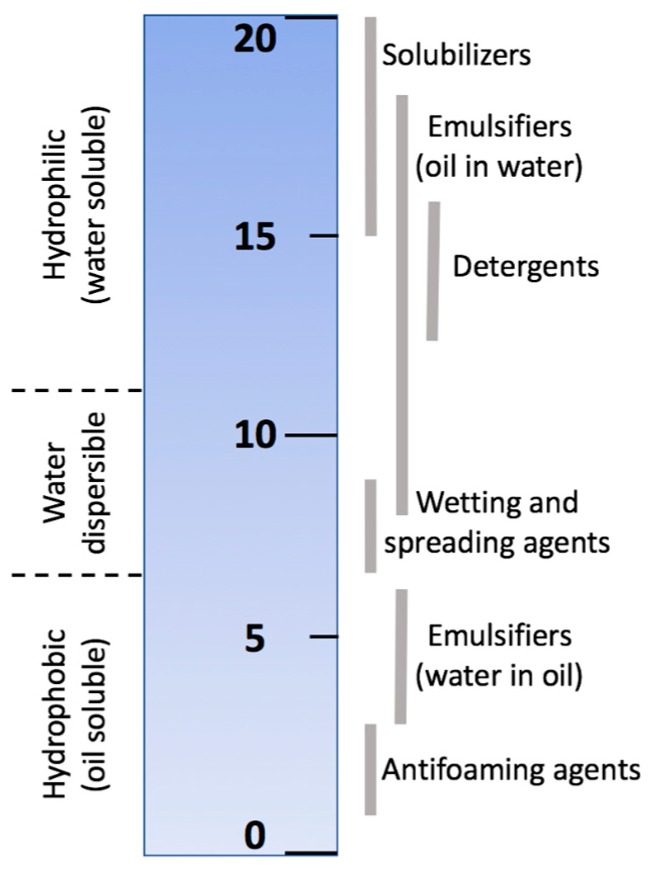
An example of the HLB scale for characterizing surfactants. Scale produced from data given in Reference [105].

Some classes of surfactants are LC (oil)-soluble and may compromise the integrity of the sensing LC material. Griffin devised an arbitrary scale for surfactant hydrophilic-lipophilic balance (HLB) [103]. This scale was defined as the ratio of the molecular weights of hydrophilic and lipophilic segments of the surfactant. Other scales were later proposed (see Figure 15) to generalize a widening range of surface-active agents. In general, the HLB value is determined by the potential for emulsion stabilization [104].

**Figure 16:**
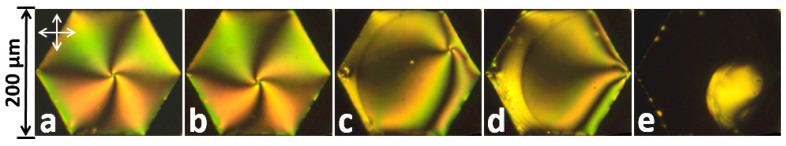
Influence of Triton-X100 on the 5CB nematic film. Surfactant concentrations in distilled water: (**a**) 0 mM; (**b**) 0.93 mM; (**c**–**e**) 0.94 mM (images taken during the process of collapse of a thin film). Vertical and horizontal arrows represent the directions of polarizer and analyzer. Reprinted from Reference [55] with permission of the Nanomaterials Research Institute of Ivanovo State University, Russia.

Surface-active materials classified as solubilizing agents, such as sodium dodecyl sulfate (SDS), are readily detected by LC cells: 5CB films normally remain stable at concentrations above those that drive the transition from hybrid into homeotropic alignment [5,47]. In contrast, Popov et al. [55] showed that 5CB-based freely-suspended nematic and cholesteric sensing films collapse in the presence of the non-ionic surfactant Triton X-100 before showing any hybrid-to-homeotropic LC realignment as shown in Figure 16:

The collapse of the LC is probably due to the formation of the LC droplets in water, since on the HLB scale Triton X-100 is classified as an emulsifier, which promotes the formation of oil-in-water emulsions. It could be very useful to design LC-based sensors considering the HLB classification of the target surface-active analyte: a popular vendor of chemicals, Sigma-Aldrich, offers surfactants that are categorized by HLB numbers [106].

### 4.2. Influence of Aqueous Solution Composition

The response of a LC-based sensor to a certain analyte may depend on the composition of the aqueous solution (buffer type): pH, ion-type and the presence of co-solutes must all be considered.

#### 4.2.1. Effect of pH

In biological sensing, an important environmental parameter is the pH value. Popov et al. [55] found that when the pH of the sub-phase was decreased with acetic acid, 5CB films become more sensitive to SDS; for example, the hybrid-to-homeotropic transition occurs at lower analyte concentrations. When the pH of the sub phase was increased with ammonium hydroxide, 5CB films became less sensitive to SDS, to the point that no hybrid-to-homeotropic transition was observed before the SDS concentration became so high that the freely suspended film collapsed. We noticed that the stability of the LC film is also dependent on the mesh size of the supporting grid. Additional investigations are required to arrive at conclusive understanding of the influence of pH level alone on the sensitivity of 5CB sensing films to various surfactants.

#### 4.2.2. Effect of Salt Type

**Figure 17:**
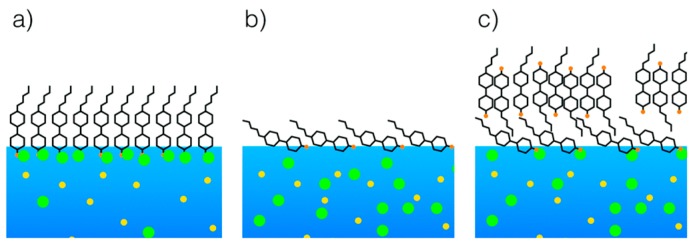
Schematic illustration of 5CB liquid crystal supported on (**a**) sodium iodide; (**b**) sodium bromide (low surface pressure) and (**c**) sodium bromide (high surface pressure). Yellow circles represent sodium ions. Green circles represent halide ions. Reproduced from [109] with permission of The Royal Society of Chemistry.

Additionally, close attention must be paid to specific anions from the Hofmeister series [107], which are reported to influence LC sensing surfaces as well as proteins [108]. As recently demonstrated by Hallett et al. [109], the Langmuir monolayer technique in conjunction with X-ray reflectivity can be used to shed light on the surface organization of cyanobiphenyl LC homologues (nCB) on aqueous solutions of different salt species. The authors found that sodium iodide tends to stabilize the monolayers of nCBs, while sodium bromide does not affect it (see Figure 17). These subtle details may play an important role in designing the next LC-based biosensors.

#### 4.2.3. Effect of co-Solute: Glycine 

**Figure 18:**
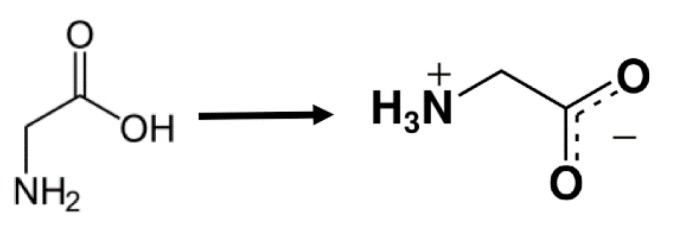
Illustration of how glycine takes a zwitterionic form in pure water.

In a recent work, we studied the change of 5CB sensitivity to SDS depending on the amount of dissolved glycine (Gly), which is the simplest possible amino acid. It plays a role as an inhibitory neurotransmitter in retina, brain and spinal cord [110]. This amino acid is particularly interesting because at typical cellular pH levels it is zwitterionic (see Figure 18) and can associate with both hydrophilic and hydrophobic environments.

**Figure 19:**
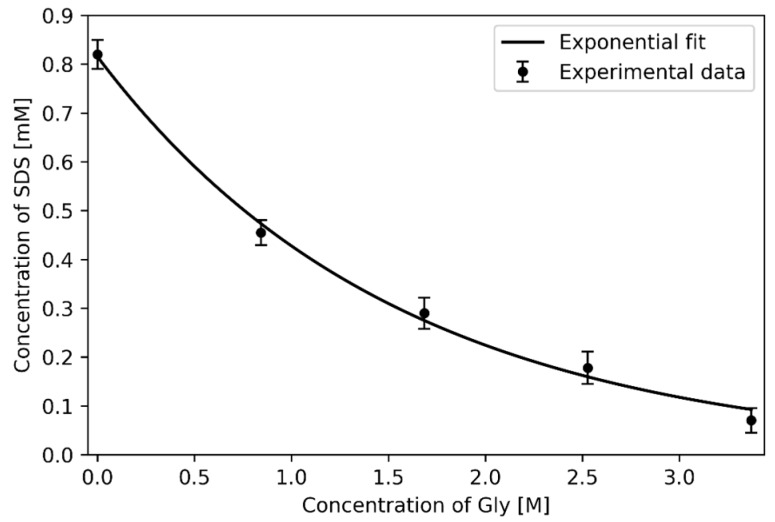
Hybrid-to-homeotropic transition of 5CB films: pH ≈ 6.5; The error bars represent the standard errors for each data point that was calculated from five independent experiments. The solubility of Gly in water at room temperature is ~3.37M.

It was found that increased Gly concentration leads to higher sensitivity of 5CB to SDS (see Figure 19). The measured pH value, was independent of Gly concentration.

**Figure 20:**
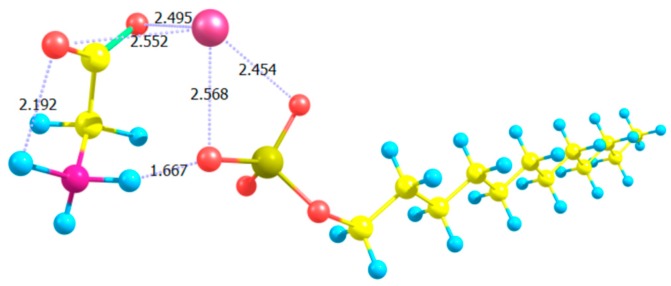
Rendering of complex formation of SDS with Gly molecules. The calculated structural model was performed in Gaussian 16 using the DFT/B97D method with basis set 6-311++G. Atoms are color-coded: carbons in yellow; hydrogens in light-blue; oxygens in red; Sulphur in pale-yellow; nitrogen in magenta; sodium counter-ion (Na^+^) in pink. Reproduced from Reference [111] with permission of the Nanomaterials Research Institute of Ivanovo State University, Russia.

To explain the effect of Gly on the sensitivity of 5CB films to SDS, we ran a simple molecular-scale simulation. We used the Gaussian 16 computational chemistry software package to model an SDS molecule in water in the presence of Gly. As shown in Figure 20, we observed that Gly molecules associate with the SDS polar head of SDS via hydrogen bonding (shown by dashed lines), effectively increasing the size of the hydrophilic head of SDS molecules, which shifts the effective HLB. As found by Usol’tseva et al. [111], Gly helps SDS form micelles at lower SDS concentrations by lowering the critical micelle concentration (CMC) value.

## 5. LC-Assisted Sensors for Specific Detection of Analytes

To date a large variety of approaches of LC assisted biological and chemical sensing have been demonstrated [7,9,17,47,55] but examples of LC-based specific sensing of analytes remain scarce [14,57,112,113]. While non-specific LC-based sensor studies are interesting and insightful, “real life” applications require highly-specific LC assisted platforms for selective sensing of analytes. Further, LC-based solutions must offer advantages compared to the currently popular specific sensor platforms such as based on surface plasmon resonance (SPR) [81], amperometric immunosensors [83], enzyme-linked immunosorbent assays (ELISA) and others [18,28]. LC-based sensor design should benefit from principles utilized in well-established techniques, as well as implement new approaches based on accumulated knowledge from fundamental research on various specific interactions between biological molecules [114,115,116].

### 5.1. Specific Biosensing

**Figure 21:**
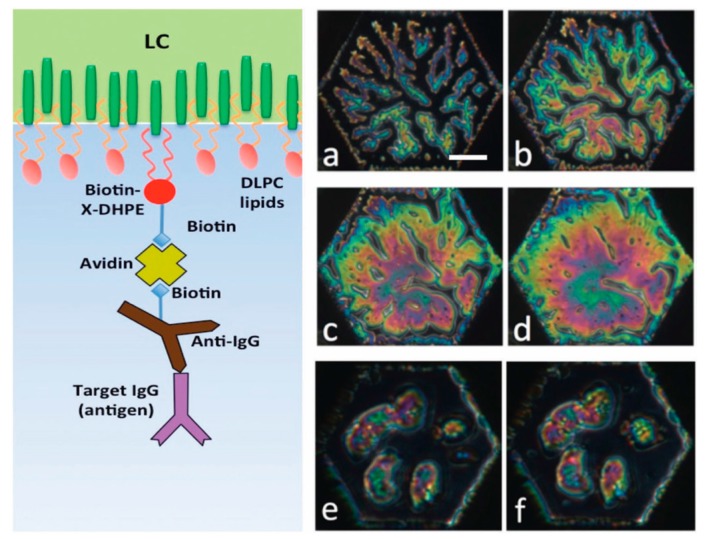
Schematic illustration of the principle of operation of an LC-based biosensor for specific detection. Left: Schematic of the decorated liquid crystal-aqueous interface. Right: Test results of prefabricated biosensor responses. Top and middle rows show the response of 5CB decorated with a monolayer of 98% DLPC and 2% Biotin-X-DHPE and biotinylated anti-goat IgG to goat IgG at 10 mg/mL: (**a**) after 20 min; (**b**) after 30 min; (**c**) after 40 min; (**d**) after 50 min. Bottom row shows the negative control experiment with rat IgGs: (**e**) after 7 min; (**f**) after 31 min. Scale bar indicates 50 µm. All textures viewed between circular polarizers. Reprinted from Reference [9].

In our recent articles [9,14], we have described our progress on the development of prefabricated biosensors that we envision could be used for providing inexpensive identification by visual confirmation of the presence of targeted antigens in the field without requiring the bulky and expensive laboratory equipment. In Figure 21, on the left side we show the schematic of the decorated LC surface, which is designed to ensure binding of target antigens out of aqueous solutions containing them. The decoration of the LC surface was performed in several steps. The details of this procedure are reported in Reference [14]. To briefly summarize, a monolayer of DLPC phospholipid molecules that contained a small amount of biotin-conjugated Biotin-X-DHPE (N-((6-(biotinoyl)amino)hexanoyl)-1,2-dihexadecanoyl-*sn*-glycero-3-phosphoethanolamine) phospholipids was self-assembled onto the LC surface. The biotin groups were then capped with neutravidin (deglycosylated avidin) molecules. Next, biotin-conjugated specific antibodies were attached to the immobilized neutravidin molecules. The responses of the functionalized LC sensor to the targeted antigen are presented in Figure 21a–d on the right side, which is manifested by the growth of the bright colored areas of the sensing hexagonal cell due to LC reorientation upon binding of antigen IgGs to the immobilized anti-IgG antibodies. The challenge in this case was to ensure that detection of the analyte led to reorientation of the LC through several intermediate layers [7,15].

**Figure 22:**
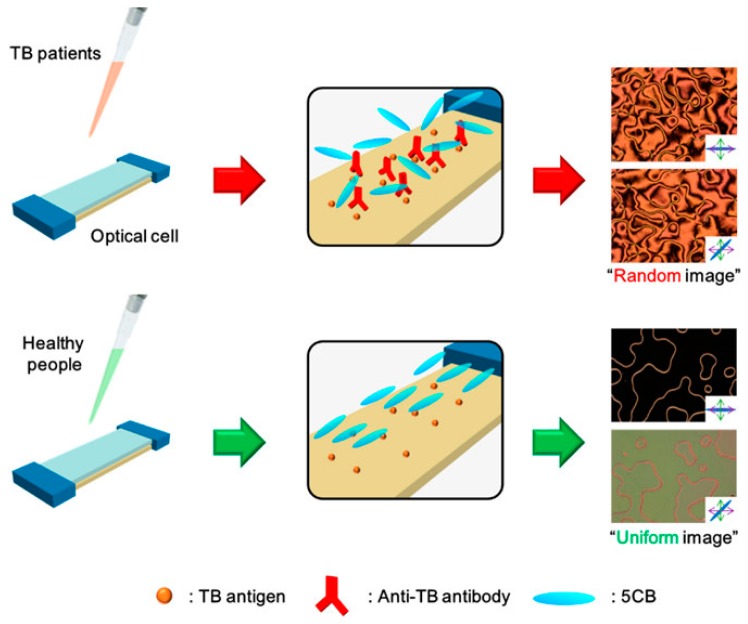
Schematic illustration of TB diagnosis in clinical specimens, using a LC-based immunosensor. With clinical serum samples from TB patients, binding of anti-TB antibodies onto a TB antigen-decorated surface causes the orientational transition of LCs, resulting in a random texture of polarized optical LC image. However, with clinical serum samples from healthy people, the orientation of LCs is not disrupted, resulting in a uniform texture of the optical LC image. Reprinted with permission from Reference [113]. Copyright 2017 American Chemical Society.

Kim et al. [113], with tuberculosis (TB) tests as an example, demonstrated that an LC-based immunosensor is a promising diagnostic platform that does not require complicated preparation of clinical specimens or complex instrumentation. As schematically shown in Figure 22, this new immunosensor for clinical TB diagnosis uses 5CB that exhibits specific orientational transitions with clinical serum samples from TB patients by antigen-antibody reactions on the TB antigen-immobilized substrate. Kim et al. determined that the frequency of false positives of the LC-based immunosensor is low (5 false positives out of 53 LC TB tests from sera of healthy people) and comparable to the results obtained from the common polymerase chain reaction (PCR) assay test for the diagnosis of TB meningitis (TBM) [117].

Shen et al. reported the development of highly specific LC-based biosensor capable of identifying complimentary ssDNA sequences and rejecting DNA even with a single-base mismatch [112]. The DNA hybridization assay was performed in an initial incubation step on a glass slide. The approaches of Shen et al. [112] and Kim et al. [113] did not capture the real-time dynamics of DNA hybridization and antibody-antigen interaction, respectively but may offer a robust technique by assembling an optical LC cell with an alignment layer formed due to specific binding of biological molecules. Additionally, increasing the stability of the sensing LC interface while maintaining the specificity requirement can be achieved by employing immobilized aptamers on the thermotropic LC surfaces that can be used to replace antibodies [18,118].

### 5.2. Specific Sensing of Gases

LC-based sensing of ammonia (NH_3_) gas was recently proposed by Niu et al. [57]. NH_3_ was detected by the disruption of the LC alignment on the glass substrate decorated with chitosan-Cu^+^, which has a higher affinity to NH_3_ than to 5CB. The authors demonstrated that this LC-based sensor is highly selective towards NH_3_, showing only a very weak response to other gases such as H_2_S, SO_2_, Cl_2_, CO_2_ and CO. Since, in this approach, the analyte is a gas diffusing into the LC sensing layer, the method captures real-time dynamics by placing the sample between a pair of polarizers. The initial dark texture, due to homeotropic alignment, turns bright as NH_3_ reaches the chitosan-Cu^+^ layer and induces random alignment. A commercial LC sensor platform for detection of NH_3,_ along with the already available LC-based sensor for the detection of hydrogen sulfide (H_2_S), has been developed by Platypus Technologies, LLC [119].

### 5.3. Other Approaches

In addition to these approaches to achieve desired specificity of LC-based sensors, other high binding affinity molecular interactions can be utilized, such as those mediated by aptamers [116,118,120,121] and enzymes [30,43,122,123]. Aptamers, unlike antibodies, are based on rigid nucleic acid backbones and are known to withstand harsh operational conditions. Aptamers can be designed in vitro, by polymerase chain reaction (PCR) through several selection rounds, to exhibit high affinity to a targeted antigen. The uniqueness of enzymes is their ability to not only recognize an analyte molecule but additionally to catalyze its transformation to specific biochemical products.

## 6. Computational Approaches to LC Sensor Designs

In recent years, huge progress has been made in the development of novel advanced functional nanomaterials. Many successful experiments have been performed in search of functional sensor designs with predetermined schemes for a reliable and informative response. Typically, the preliminary design ideas are quite simple but very often unexpected behaviors result, especially when the analytes are complex biological molecules. Clearly, optimizing LC sensing devices requires tracking chemical reactions and physical interactions on an atomic level. Thus, systematic computational chemistry and molecular dynamics methods may prove to be very useful approaches.

### 6.1. Computational Chemistry Methods

**Figure 23:**
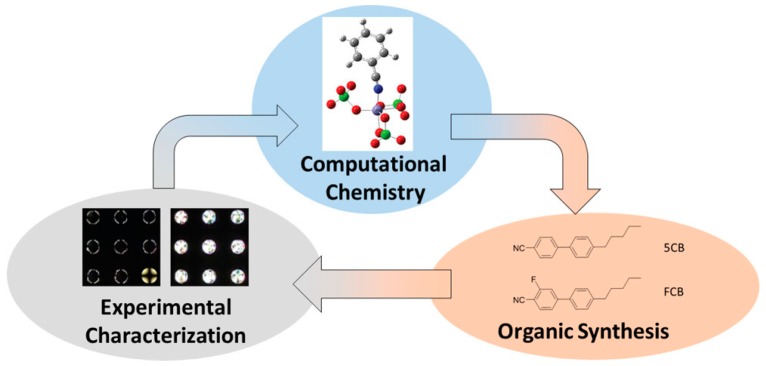
General flow chart of computational chemistry methods for designing chemically responsive LCs. Reprinted with permission from Reference [124]. Copyright 2017 American Chemical Society.

Optimization of a particular LC sensor platform is often a rather challenging task due to an involvement of a high number of experimental parameters, some of them not even identified in initial experimental trials. To address this challenge, Szilvási et al. [124] proposed an approach of integrating cycles of computational chemistry, organic synthesis and physical property evaluation for the efficient design of novel chemo-responsive LCs as schematically represented in Figure 23:

In this work, Szilvási et al. used electronic structure calculations of the binding of nitrile-containing mesogens to perchlorate salts. This approach predicted that selective fluorination can reduce the strength of binding of nitrile-containing nematic LCs to metal-salt decorated surfaces and thus generate a faster reordering of the LC in response to competitive binding of dimethyl methylphosphonate (DMMP) gas. The authors subsequently synthesized and tested several fluorinated compounds and confirmed their theoretical predictions of their “coordinately saturated anion model” (CSAM). In this model, the perchlorate salt ion is explicitly described since, as shown in [125], the choice of the salt anion can influence the interaction of nitrile-containing mesogens with metal-salt decorated surfaces. The CSAM model showed that selective fluorination of the 5CB mesogens reduces the binding energy of the nitrile group to metal cations and thus leads to a substantial response to DMMP.

### 6.2. Molecular Dynamics

In recent years, huge progress has been made in the development of novel advanced functional nanomaterials. Many successful experiments have been performed in search of functional sensor designs with predetermined schemes for a reliable and informative response. Typically, the preliminary design ideas are quite simple but very often unexpected behaviors result, especially when the analytes are complex biological molecules. Clearly, optimizing LC sensing devices requires tracking chemical reactions and physical interactions on an atomic level and thus systematic computational chemistry and molecular dynamics methods may become very useful tools.

Atomistic Molecular Dynamics (MD) calculations often require huge computer resources, even in the case of optimized and parallelized simulated systems, which typically need to contain a very large number of interacting molecules to properly describe realistic physical environments and events. One way of simulating large biological systems is to depart from the atomistic level of details and simplify the structure of molecules by, for example, representing phospholipids, proteins and other relevant molecules in coarse-grained (CG) simplification [126,127,128] and reducing the rod-like LC molecules to spheroids [129].

**Figure 24:**
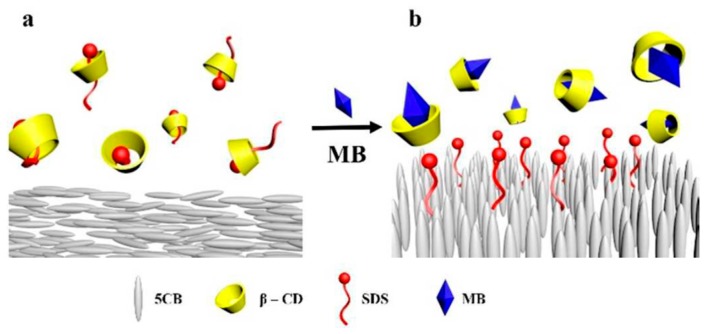
MD simulations of competitive guest-host interaction of β-CD (β-cyclodextrin) and MB (methylene blue) applied in an air-supported LC sensor. (**a**) Planar alignment of 5CB; (**b**) homeotropic alignment of 5CB. Reproduced from Reference [139] with permission from the PCCP Owner Societies.

Luckily, the famous Moore’s “law,” which predicts the computational power of computers doubles approximately every 18 months, is still in effect. Thus, simulations are expected to be effective for increasingly more complex systems. Larger number of atoms and longer simulation time frames become accessible to reproduce realistic binding/unbinding events that may occur in biosensor systems [120,121,130,131,132,133]. Recently, complete fully atomistic models of capsids such as of HIV-1 and Hepatitis-B viruses were developed [134,135,136], which may facilitate much more accurate modeling of interaction events at biosensor/capsid interfaces. MD simulations were also employed in revealing the details of the organization of LC molecules in thin films at interfaces [137,138,139] (See Figure 24) and were used in predicting their stability and rupture mechanisms [140]. When simulations results agree well with real experimental observations, the MD serves as a “computational microscope” [141] that allows visualization of objects that often no other tool can.

The realism of MD simulations is highly dependent on the accuracy of the chosen force field (FF). Many FFs were developed in the last decades but perhaps the most applicable to modeling of LC biosensors is the FF named Optimized Potentials for Liquid Systems (OPLS). Incremental updates to this FF allowed in recent years the more accurate reproduction of phenomena occurring at liquid interfaces [142]. An improved version OPLS3 is now available. As reported by Schrödinger, LLC (New York, NY, USA) [143], this new version adequately covers the medicinal-chemical space by employing over an order of magnitude more reference data and associated parameter types relative to other commonly-used small molecule force fields (e.g. MMFF, OPLS_2005, etc.). Electronic coarse-graining is another important approach for more realistic reproduction of biologically-relevant forces such as many-body polarization and dispersions [144].

LC biosensors were reported to identify enzymatic reactions [145,146]. Such chemical reactions cannot be simulated with conventional FFs. Thus, recently a new reactive force field (ReaxFF) was developed [147,148]. Improvements to QM/MM (quantum mechanics/molecular mechanics) methods in multi-scale modeling were reported as well [149]. Development of such emerging MD tools [150] is crucial for accessing the full range of possible biochemical events for the optimal design of sensors, drug-delivery systems, immunoresponse manipulation and so on.

## 7. Summary and Future Directions

The field of chemical and biological sensing using thermotropic LCs has made great progress during the 20 years since the introduction, by Gupta et al. in 1998 [15], of the method of optical amplification of ligand-receptor binding using LCs. Many sensing platform modifications and improvements have been suggested since then. Many milestones have been reached and reported in numerous articles [7,9]. 

Research achievements strongly suggest that the LC approach is indeed a very promising one but further challenges in the development of functional chemical and biological sensors that will be competitive with solutions already on the market need to be addressed. These include:

- Sensing LC interfaces need to be robust. This is especially important for platforms with flat sensing surfaces, in which LC films need to be protected from rupture or becoming washed away into the aqueous solution [17,55,140].
- Detection limits need to be as low as or lower than the sensitivities of existing non-LC sensors. Additionally, a linear sensor response over the interesting range is desirable [6].
- New types of detection modes that are not based on measurements performed at often slowly-achieved equilibrium states, may become very useful. Further, the final equilibrium states induced by different analytes may appear similar and even indistinguishable but the dynamics in achieving that final equilibrium state may reveal the presence of a specific analyte. Thus, non-equilibrium processes deserve serious attention in the future development of LC-assisted sensor platforms [5].
- One of the most important of customer requirements is that the sensors need to be extremely specific towards a single kind of analyte of interest. Research that demonstrates true and reliable specificity of LC-assisted sensors employing antibodies, aptamers and similar molecules remains scarce [14]. Reproducing and simplifying the biomimetic mechanisms of molecular recognition may serve as an efficient way of ensuring the required high specificity of biosensors [36].
- Another important market requirement is that the final sensor platform must be compact and especially easy and ready to use without much preparation. Thus, prefabricated devices with long shelf-lives are in high demand. At least two possible directions have been suggested: Ionic LCs may prevent fragile enzymes and antibodies from denaturing [99]. Aptamers, much more stable than antibodies, may provide the required level of specificity [121].
- Fundamental research on the behavior of LC-based sensors towards specific analytes in the presence of interfering species [55,108] is only at the beginning. This extremely important problem will need to be extensively addressed prior to any serious attempts at commercializing new types of sensors. It is likely that LC/analyte interactions, as well as chemical reactions, will need to be clearly understood and visualized at the molecular level for the successful development of superior future sensors [124,139].

In this review, we have summarized the most recent progress on the development of novel LC-based sensors for chemical and biological analytes. Even though this field is still in its infancy, sensor technologies revolving around LC materials are being patented and commercialized [37,119]. On a broader scale, we recognize that novel platforms (not necessarily LC-based) for specific, reliable, inexpensive and rapid sensing are in growing demand [151]. These needs are validated by various companies such as ProXentia S.r.l. (Milan, Lombardy, Italy) [152] and Dynamic Biosensors Inc. (Martinsried, Munich, Bavaria, Germany) [153] and many others [154], entering this market. Due to the dramatic responsiveness of LCs to various stimuli [155], LC-based solutions hold the promise for significant breakthroughs in sensor applications. For biological applications, LC materials deserve additional attention due to the omnipresence of LC phases in various developing tissues of the brain, liver, kidneys and other organs [156]. All these unique and exciting features of LCs provide a myriad of opportunities for the development of novel smart functional materials and place these self-organizing substances at the forefront of ongoing global research.

## References

[1] Palffy-Muhoray P. (2007). The diverse world of liquid crystals. Phys. Today.

[2] Li Q. (2012). Liquid Crystals Beyond Displays.

[3] Van der Asdonk P., Kouwer P.H.J. (2017). Liquid crystal templating as an approach to spatially and temporally organise soft matter. Chem. Soc. Rev..

[4] Schenning A., Crafword G.P., Broer D.J. (2017). Liquid Crystal Sensors.

[5] Popov P., Mann E.K., Jákli A. (2014). Accurate Optical Detection of Amphiphiles at Liquid-Crystal-Water Interfaces. Phys. Rev. Appl..

[6] Iglesias W., Abbott N.L., Mann E.K., Jákli A. (2012). Improving liquid-crystal-based biosensing in aqueous phases. ACS Appl. Mater. Interfaces.

[7] Carlton R.J., Hunter J.T., Miller D.S., Abbasi R., Mushenheim P.C., Tan L.N., Abbott N.L. (2013). Chemical and biological sensing using liquid crystals. Liq. Cryst. Rev..

[8] Tan L.N., Carlton R.J., Cleaver K., Abbott N.L. (2014). Liquid Crystal-Based Sensors for Rapid Analysis of Fatty Acid Contamination in Biodiesel. Mol. Cryst. Liq. Cryst..

[9] Popov P., Mann E.K., Jakli A. (2017). Thermotropic liquid crystal films for biosensors and beyond. J. Mater. Chem. B.

[10] Hunter J.T., Abbott N.L. (2013). Dynamics of the chemo-optical response of supported films of nematic liquid crystals. Sens. Actuators B Chem..

[11] Reyes C.G., Sharma A., Lagerwall J.P.F. (2016). Non-electronic gas sensors from electrospun mats of liquid crystal core fibres for detecting volatile organic compounds at room temperature. Liq. Cryst..

[12] Shibaev P.V., Wenzlick M., Murray J., Tantillo A., Howard-Jennings J. (2015). Rebirth of Liquid Crystals for Sensoric Applications: Environmental and Gas Sensors. Adv. Condens. Matter Phys..

[13] Hussain A., Semeano A.T.S., Palma S.I.C.J., Pina A.S., Almeida J., Medrado B.F., Pádua A.C.C.S., Carvalho A.L., Dionísio M., Li R.W.C. (2017). Tunable Gas Sensing Gels by Cooperative Assembly. Adv. Funct. Mater..

[14] Popov P., Honaker L.W., Kooijman E.E., Mann E.K., Jákli A.I. (2016). A liquid crystal biosensor for specific detection of antigens. Sens. Bio-Sens. Res..

[15] Gupta V.K., Skaife J.J., Dubrovsky T.B., Abbott N.L. (1998). Optical Amplification of Ligand-Receptor Binding Using Liquid Crystals. Science.

[16] Brake J.M., Abbott N.L. (2007). Coupling of the orientations of thermotropic liquid crystals to protein binding events at lipid-decorated interfaces. Langmuir.

[17] Popov P. (2015). Liquid Crystal Interfaces: Experiments, Simulations and Biosensors. http://rave.ohiolink.edu/etdc/view?acc_num=kent1434926908.

[18] Walker J.M. (2017). Biosensors and Biodetection.

[19] Narayan R.J. (2016). Medical Biosensors for Point of Care (POC) Applications.

[20] Urban G. (2013). Applications of Nanomaterials in Sensors and Diagnostics.

[21] Ahmed M.U., Saaem I., Wu P.C., Brown A.S. (2014). Personalized diagnostics and biosensors: A review of the biology and technology needed for personalized medicine. Crit. Rev. Biotechnol..

[22] Hasan A., Nurunnabi M., Morshed M., Paul A., Polini A., Kuila T., Al Hariri M., Lee Y., Jaffa A.A. (2014). Recent Advances in Application of Biosensors in Tissue Engineering. Biomed. Res. Int..

[23] Wu H., Shang Y., Zhang J., Cheang L.H., Zeng X., Tu M. (2017). The effects of liquid crystal-based composite substrates on cell functional responses of human umbilical cord-derived mesenchymal stem cells by mechano-regulatory process. J. Biomater. Appl..

[24] Ünlü N.L., Kanik F.E., Seymour E., Connor J.H., Ünlü M.S., Walker J.M. (2017). Optical-Based Detectors. Biosensors and Biodetection.

[25] Fang Y. (2006). Label-Free Cell-Based Assays with Optical Biosensors in Drug Discovery. Assay Drug Dev. Technol..

[26] Cooper M.A. (2006). Optical biosensors: Where next and how soon?. Drug Discov. Today.

[27] Gavela A.F., García D.G., Ramirez J., Lechuga L. (2016). Last Advances in Silicon-Based Optical Biosensors. Sensors.

[28] Zanchetta G., Lanfranco R., Giavazzi F., Bellini T., Buscaglia M. (2017). Emerging applications of label-free optical biosensors. Nanophotonics.

[29] Hussain Z., Qazi F., Ahmed M.I., Usman A., Riaz A., Abbasi A.D. (2016). Liquid crystals based sensing platform-technological aspects. Biosens. Bioelectron..

[30] Setia S., Sidiq S., De J., Pani I., Pal S.K. (2016). Applications of liquid crystals in biosensing and organic light-emitting devices: Future aspects. Liq. Cryst..

[31] Wang D., Park S.-Y., Kang I.-K. (2015). Liquid crystals: Emerging materials for use in real-time detection applications. J. Mater. Chem. C..

[32] Munir S., Kang I.-K., Park S.-Y. (2016). Polyelectrolytes functionalized nematic liquid crystal-based biosensors: An overview. TrAC Trends Anal. Chem..

[33] Van’t Hag L., Gras S.L., Conn C.E., Drummond C.J. (2017). Lyotropic liquid crystal engineering moving beyond binary compositional space—Ordered nanostructured amphiphile self-assembly materials by design. Chem. Soc. Rev..

[34] Zhou S. (2017). Lyotropic Chromonic Liquid Crystals.

[35] Shiyanovskii S.V., Lavrentovich O.D., Schneider T., Ishikawa T., Smalyukh I.I., Woolverton C.J., Niehaus G.D., Doane K.J. (2005). Lyotropic Chromonic Liquid Crystals for Biological Sensing Applications. Mol. Cryst. Liq. Cryst..

[36] De Souza J., Pontes K., Alves T., Amaral V., Rebelo M., Hausen M., Chaud M. (2017). Spotlight on Biomimetic Systems Based on Lyotropic Liquid Crystal. Molecules.

[37] Crystal Diagnostics LTD, (n.d.). http://www.crystaldiagnostics.com/.

[38] Van der Asdonk P., Collings P.J., Kouwer P.H.J. (2017). Fully Stable and Homogeneous Lyotropic Liquid Crystal Alignment on Anisotropic Surfaces. Adv. Funct. Mater..

[39] Berride F., Troche E., Feio G., Cabrita E., Sierra T., Azquez A.N.V., Cid M. (2017). Chiral amplification of disodium cromoglycate chromonics induced by a codeine derivative. Soft Matter.

[40] Salamonczyk M., Zhang J., Portale G., Zhu C., Kentzinger E., Gleeson J.T., Jakli A., de Michele C., Dhont J.K.G., Sprunt S. (2016). Smectic phase in suspensions of gapped DNA duplexes. Nat. Commun..

[41] Bellini T., Zanchetta G., Fraccia T.P., Cerbino R., Tsai E., Smith G.P., Moran M.J., Walba D.M., Clark N.A. (2012). Liquid crystal self-assembly of random-sequence DNA oligomers. Proc. Natl. Acad. Sci. USA.

[42] Barbero G., Evangelista L.R. (2014). Adsorption phenomena and anchoring energy in nematic liquid crystals. Liq. Cryst. Rev..

[43] Kim H.J., Jang C. (2016). Micro-capillary sensor for imaging trypsin activity using confined nematic liquid crystals. J. Mol. Liq..

[44] Kim H.J., Jang C.-H. (2017). Liquid crystal-based capillary sensory platform for the detection of bile acids. Chem. Phys. Lipids.

[45] Enz E., La Ferrara V., Scalia G. (2013). Confinement-Sensitive Optical Response of Cholesteric Liquid Crystals in Electrospun Fibers. ACS Nano.

[46] Noh J.H., Henx B., Lagerwall J.P.F. (2016). Taming Liquid Crystal Self-Assembly: The Multifaceted Response of Nematic and Smectic Shells to Polymerization. Adv. Mater..

[47] Jakli A., Mann E.K., Popov P. (2015). System and Method Thereof for Accurate Optical Detection of Amphiphiles at a Liquid Crystal Interface. http://www.freepatentsonline.com/y2015/0233816.html.

[48] Lee M.-J., Sung Y.-C., Hsiao Y.-C., Lee W. (2016). Chiral liquid crystals as biosensing platforms. Proc. SPIE.

[49] Jang J.H., Park S.Y. (2017). pH-responsive cholesteric liquid crystal double emulsion droplets prepared by microfluidics. Sens. Actuators B Chem..

[50] Lee H.-G., Munir S., Park S.-Y. (2016). Cholesteric Liquid Crystal Droplets for Biosensors. ACS Appl. Mater. Interfaces.

[51] Lee M.-J., Chang C.-H., Lee W. (2017). Label-free protein sensing by employing blue phase liquid crystal. Biomed. Opt. Express.

[52] Bukusoglu E., Martinez-Gonzalez J.A., Wang X., Zhou Y., de Pablo J.J., Abbott N.L. (2017). Strain-induced alignment and phase behavior of blue phase liquid crystals confined to thin films. Soft Matter.

[53] Hartono D., Bi X., Yang K.-L., Yung L.-Y.L. (2008). An Air-Supported Liquid Crystal System for Real-Time and Label-Free Characterization of Phospholipases and Their Inhibitors. Adv. Funct. Mater..

[54] Popov P., Mann E.K., Jakli A. (2014). Liquid-Crystal-Based Biosensor without Alignment Substrate. Biophys. J..

[55] Popov N., Smirnova A., Usol’tseva N., Popov P. (2017). Determination of Concentrations of Surface-Active Materials in Aqueous Solutions at Different pH Values Using Liquid Crystals. Liq. Cryst. Their Appl..

[56] Popov P., Honaker L.W., Mirheydari M., Mann E.K., Jákli A. (2017). Chiral nematic liquid crystal microlenses. Sci. Rep..

[57] Niu X., Zhong Y., Chen R., Wang F., Luo D. (2017). Highly sensitive and selective liquid crystal optical sensor for detection of ammonia. Opt. Express.

[58] Pantoja M.A.B., Abbott N.L. (2016). Surface-Controlled Orientational Transitions in Elastically Strained Films of Liquid Crystal That Are Triggered by Vapors of Toluene. ACS Appl. Mater. Interfaces.

[59] Wang X., Zhou Y., Kim Y.K., Miller D.S., Zhang R., Martinez-Gonzalez J.A., Bukusoglu E., Zhang B., Brown T.M., de Pablo J.J. (2017). Patterned surface anchoring of nematic droplets at miscible liquid–liquid interfaces. Soft Matter.

[60] Tran L., Lavrentovich M.O., Durey G., Darmon A., Haase M.F., Li N., Lee D., Stebe K.J., Kamien R.D., Lopez-Leon T. (2017). Change in Stripes for Cholesteric Shells via Anchoring in Moderation. Phys. Rev. X.

[61] Noh J., de Sousa K.R., Lagerwall J.P.F. (2015). Influence of interface stabilisers and surrounding aqueous phases on nematic liquid crystal shells. Soft Matter.

[62] Shah R.K., Shum H.C., Rowat A.C., Lee D., Agresti J.J., Utada A.S., Chu L., Kim J., Fernandez-nieves A., Martinez C.J. (2008). Designer emulsions using microfluidics. Mater. Today.

[63] Brosseau Q., Vrignon J., Baret J.-C. (2014). Microfluidic Dynamic Interfacial Tensiometry (μDIT). Soft Matter.

[64] Tang S.Y., Joshipura I.D., Lin Y., Kalantar-Zadeh K., Mitchell A., Khoshmanesh K., Dickey M.D. (2016). Liquid-Metal Microdroplets Formed Dynamically with Electrical Control of Size and Rate. Adv. Mater..

[65] Humar M., Muševič I. (2011). Surfactant sensing based on whispering-gallery-mode lasing in liquid-crystal microdroplets. Opt. Express.

[66] Buyuktanir E.A., Frey M.W., West J.L. (2010). Self-assembled, optically responsive nematic liquid crystal/polymer core-shell fibers: Formation and characterization. Polymer.

[67] West J.L., Wang J., Jákli A. (2017). Airbrushed Liquid Crystal/Polymer Fibers for Responsive Textiles. Adv. Sci. Technol..

[68] Wang J., West J.L. (2016). Morphology Tuning of Electrospun Liquid Crystal/Polymer Fibers. Chemphyschem.

[69] Wang J., Kolacz J., Chen Y., Jákli A., Kawalec J., Benitez M., West J.L. (2017). Smart Fabrics Functionalized by Liquid Crystals. J. Soc. Inf. Disp..

[70] Kye Y., Kim C., Lagerwall J. (2015). Multifunctional responsive fibers produced by dual liquid crystal core electrospinning. J. Mater. Chem. C.

[71] Kim D.K., Hwang M., Lagerwall J.P.F. (2013). Liquid crystal functionalization of electrospun polymer fibers. J. Polym. Sci. Part B Polym. Phys..

[72] Urbanski M., Reyes C.G., Noh J., Sharma A., Geng Y., Jampani V.S.R., Lagerwall J.P.F. (2017). Liquid crystals in micron-scale droplets, shells and fibers. J. Phys. Condens. Matter.

[73] Georgakilas V., Tiwari J.N., Kemp K.C., Perman J.A., Bourlinos A.B., Kim K.S., Zboril R. (2016). Noncovalent Functionalization of Graphene and Graphene Oxide for Energy Materials, Biosensing, Catalytic, and Biomedical Applications. Chem. Rev..

[74] Wei Y., Jang C.H. (2017). Liquid crystal as sensing platforms for determining the effect of graphene oxide-based materials on phospholipid membranes and monitoring antibacterial activity. Sens. Actuators B Chem..

[75] Kim D.W., Kim Y.H., Jeong H.S., Jung H.T. (2011). Direct visualization of large-area graphene domains and boundaries by optical birefringency. Nat. Nanotechnol..

[76] Shehzad M.A., Tien D.H., Iqbal M.W., Eom J., Park J.H., Hwang C., Seo Y. (2015). Nematic Liquid Crystal on a Two Dimensional Hexagonal Lattice and its Application. Sci. Rep..

[77] Shehzad M.A., Hussain S., Lee J., Jung J., Lee N., Kim G., Seo Y. (2017). Study of Grains and Boundaries of Molybdenum Diselenide and Tungsten Diselenide Using Liquid Crystal. Nano Lett..

[78] Lim Y.J., Lee B.H., Kwon Y.R., Choi Y.E., Murali G., Lee J.H., Nguyen V.L., Lee Y.H., Lee S.H. (2015). Monitoring defects on monolayer graphene using nematic liquid crystals. Opt. Express.

[79] Basu R., Kinnamon D., Garvey A. (2015). Detection of graphene chirality using achiral liquid crystalline platforms. J. Appl. Phys..

[80] Basu R., Kinnamon D., Garvey A. (2016). Graphene and liquid crystal mediated interactions. Liq. Cryst..

[81] Zhang X.L., Liu Y., Fan T., Hu N., Yang Z., Chen X., Wang Z.-Y., Yang J. (2017). Design and Performance of a Portable and Multichannel SPR Device. Sensors.

[82] Lynn N.S., Sipova H., Adam P., Homola J. (2013). Enhancement of affinity-based biosensors: Effect of sensing chamber geometry on sensitivity. Lab Chip.

[83] Tomassetti M., Merola G., Martini E., Campanella L., Sanzò G., Favero G., Mazzei F. (2017). Comparison between a Direct-Flow SPR Immunosensor for Ampicillin and a Competitive Conventional Amperometric Device: Analytical Features and Possible Applications to Real Samples. Sensors.

[84] Larson R.G. (1999). The Structure and Rheology of Complex Fluids, OUP USA. https://books.google.com/books?id=Vt9fw_pf1LUC.

[85] Abuabed A. (2017). Study of the Effect of Nematic Order Degradation in Liquid Crystal-Based Surface Plasmon Resonance Sensors. Photonics.

[86] Wu P.-C., Karn A., Lee M.-J., Lee W., Chen C.-Y. (2018). Dye-liquid-crystal-based biosensing for quantitative protein assay. Dyes Pigment.

[87] Popova M., Bretz S.L., Hartley C.S. (2016). Visualizing Molecular Chirality in the Organic Chemistry Laboratory Using Cholesteric Liquid Crystals. J. Chem. Educ..

[88] Mulder D.J., Schenning A.P.H.J., Bastiaansen C.W.M. (2014). Chiral-nematic liquid crystals as one dimensional photonic materials in optical sensors. J. Mater. Chem. C.

[89] Matsumura K., Opiekun M., Oka H., Vachani A., Albelda S.M., Yamazaki K., Beauchamp G.K. (2009). Urinary volatile compounds as biomarkers for lung cancer: A proof of principle study using odor signatures in mouse models of lung cancer. PLoS ONE.

[90] Sponring A., Filipiak W., Mikoviny T., Ager C., Schubert J., Miekisch W., Amann A., Troppmair J. (2009). Release of volatile organic compounds from the lung cancer cell line NCI-H2087 in vitro. Anticancer Res..

[91] Filipiak W., Sponring A., Mikoviny T., Ager C., Schubert J., Miekisch W., Amann A., Troppmair J. (2008). Release of volatile organic compounds (VOCs) from the lung cancer cell line CALU-1 in vitro. Cancer Cell Int..

[92] Minh T.D.C., Blake D.R., Galassetti P.R. (2012). The clinical potential of exhaled breath analysis for diabetes mellitus. Diabetes Res. Clin. Pract..

[93] Van Delden R.A., Feringa B.L. (2002). Colour indicator for enantiomeric excess and assignment of the configuration of the major enantiomer of an amino acid ester. Chem. Commun..

[94] Eelkema R., Feringa B.L. (2006). Amplification of chirality in liquid crystals. Org. Biomol. Chem..

[95] Kek K.J., Lee J.J.Z., Otono Y., Ishihara S. (2017). Chemical gas sensors using chiral nematic liquid crystals and its application. J. Soc. Inf. Disp..

[96] Otono Y., Kek K.J., Jia J., Lee Z., Ishihara S., Nakano Y., Hashimotodani K., Oka H. (2016). Chemical gas sensors using chiral nematic liquid crystals. J. Soc. Inf. Disp..

[97] Eimura H., Miller D.S., Wang X., Abbott N.L., Kato T. (2016). Self-Assembly of Bioconjugated Amphiphilic Mesogens Having Specific Binding Moieties at Aqueous-Liquid Crystal Interfaces. Chem. Mater..

[98] Munir S., Park S. (2018). Liquid-crystal droplets functionalized with a non-enzymatic moiety for glucose sensing. Sens. Actuators B Chem..

[99] Fernandez A.A., Kouwer P. (2016). Key Developments in Ionic Liquid Crystals. Int. J. Mol. Sci..

[100] Atta N., BinSabt M., Hassan S., Galal A. (2017). Ionic Liquid Crystals Modifier for Selective Determination of Terazosin Antihypertensive Drug in Presence of Common Interference Compounds. Crystals.

[101] Toledo Hijo A.A., Maximo G.J., Costa M.C., Cunha R.L., Pereira J.F., Kurnia K.A., Batista E.A., Meirelles A.J. (2017). Phase Behavior and Physical Properties of New Biobased Ionic Liquid Crystals. J. Phys. Chem. B.

[102] Munje R.D., Muthukumar S., Jagannath B., Prasad S. (2017). A new paradigm in sweat based wearable diagnostics biosensors using Room Temperature Ionic Liquids (RTILs). Sci. Rep..

[103] Griffin W.C. (1949). Classification of surface-active agents by“ HLB”. J. Soc. Cosmet. Chem..

[104] Kale S.N., Deore S.L. (2016). Emulsion Micro Emulsion and Nano Emulsion: A Review. Syst. Rev. Pharm..

[105] Ansel H.C., Popovich N.G., Allen L.V. (2013). Disperse Systems. Ansel’s Pharmaceutical Dosage Forms and Drug Delivery Systems.

[106] Sigma-Aldrich. Surfactants Classified by HLB Numbers, (n.d.). http://www.sigmaaldrich.com/materials-science/material-science-products.html?TablePage=22686648%0D.

[107] Senske M., Constantinescu-Aruxandei D., Havenith M., Herrmann C., Rtner H.W., Ebbinghaus S. (2016). The temperature dependence of the Hofmeister series: Thermodynamic fingerprints of cosolute-protein interactions. Phys. Chem. Chem. Phys..

[108] Carlton R.J., Ma C.D., Gupta J.K., Abbott N.L. (2012). Influence of specific anions on the orientational ordering of thermotropic liquid crystals at aqueous interfaces. Langmuir.

[109] Hallett J.E., Hayward D.W., Arnold T., Bartlett P., Richardson R.M. (2017). X-ray reflectivity reveals ionic structure at liquid crystal–aqueous interfaces. Soft Matter.

[110] Yao L., Zhou Q. (2017). Enhancing NMDA Receptor Function: Recent Progress on Allosteric Modulators. Neural Plast..

[111] Usol’tseva N.V., Smirnova А.I., Zharnikova N.V., Kurbatova M.S., Giricheva N.I., Badelin V.G. (2016). Effect of Glycine on Lyomesophase Formation by Sodium Dodecylsulfate—Water Systems. Liq. Cryst. Their Appl..

[112] Shen J., He F., Chen L., Ding L., Liu H., Wang Y., Xiong X. (2017). Liquid crystal-based detection of DNA hybridization using surface immobilized single-stranded DNA. Microchim. Acta.

[113] Kim H.J., Rim J., Jang C.-H. (2017). Liquid-Crystal-Based Immunosensor for Diagnosis of Tuberculosis in Clinical Specimens. ACS Appl. Mater. Interfaces.

[114] Chambers J.P., Arulanandam B.P., Matta L.L., Weis A., Valdes J.J. (2008). Biosensor recognition elements. Curr. Issues Mol. Biol..

[115] Eni-olorunda I., Sadana A., Hopkins N.A.E., Meyer S.C., Ghosh I., Baldrich E., Herold K.E., Rasooly A., Laurenceau E., Lim S. (2010). Recognition Receptors in Biosensors.

[116] Lim S.A., Ahmed M.U. (2017). Chapter 1. Introduction to Food Biosensors. Food Biosensors.

[117] Deshpande P.S., Kashyap R.S., Ramteke S.S., Nagdev K.J., Purohit H.J., Taori G.M., Daginawala H.F. (2007). Evaluation of the IS6110 PCR assay for the rapid diagnosis of tuberculous meningitis. Cerebrospinal Fluid Res..

[118] Zhao D., Peng Y., Xu L., Zhou W., Wang Q., Guo L. (2015). Liquid-Crystal Biosensor Based on Nickel-Nanosphere-Induced Homeotropic Alignment for the Amplified Detection of Thrombin. ACS Appl. Mater. Interfaces.

[119] Platypus Technologies, LLC, (n.d.). http://www.platypustech.com/dosimeters.

[120] Jeddi I., Saiz L. (2017). Three-dimensional modeling of single stranded DNA hairpins for aptamer-based biosensors. Sci. Rep..

[121] Ruan M., Seydou M., Noel V., Piro B., Maurel F., Barbault F. (2017). Molecular Dynamics Simulation of a RNA Aptasensor. J. Phys. Chem. B.

[122] Das D., Pal S.K. (2017). Liquid Crystal Unveiled Interactions between Melittin and Phospholipids at Aqueous-Liquid Crystal Interface. ChemistrySelect.

[123] Munir S., Khan M., Park S.-Y. (2015). Bienzyme liquid-crystal-based cholesterol biosensor. Sens. Actuators B Chem..

[124] Szilvási T., Roling L.T., Yu H., Rai P., Choi S., Twieg R.J., Mavrikakis M., Abbott N.L. (2017). Design of Chemoresponsive Liquid Crystals through Integration of Computational Chemistry and Experimental Studies. Chem. Mater..

[125] Hunter J.T., Abbott N.L. (2014). Adsorbate-Induced Anchoring Transitions of Liquid Crystals on Surfaces Presenting Metal Salts with Mixed Anions. ACS Appl. Mater. Interfaces.

[126] Orellana L., Yoluk O., Carrillo O., Orozco M., Lindahl E. (2016). Prediction and validation of protein intermediate states from structurally rich ensembles and coarse-grained simulations. Nat. Commun..

[127] Poma A.B., Cieplak M., Theodorakis P.E. (2017). Combining the MARTINI and Structure-Based Coarse-Grained Approaches for the Molecular Dynamics Studies of Conformational Transitions in Proteins. J. Chem. Theory Comput..

[128] Niesen M.J.M., Wang C.Y., van Lehn R.C., Miller T.F. (2017). Structurally detailed coarse-grained model for Sec-facilitated co-translational protein translocation and membrane integration. PLoS Comput. Biol..

[129] Ohadi D., Uline M.J. (2017). Molecular Modeling of Liquid Crystal/Phospholipid Interface as a Label-Free Biosensor. Biophys. J..

[130] Zhang W., Du Y., Cranford S.W., Wang M.L. (2016). Biosensor Design through Molecular Dynamics Simulation. Int. J. Biol. Biomol. Agric. Food Biotechnol. Eng..

[131] Wang Y., Cai W., Chen L., Wang G. (2017). Molecular dynamics simulation reveals how phosphorylation of tyrosine 26 of phosphoglycerate mutase 1 upregulates glycolysis and promotes tumor growth. Oncotarget.

[132] Okimoto N., Suenaga A., Taiji M. (2016). Evaluation of protein–ligand affinity prediction using steered molecular dynamics simulations. J. Biomol. Struct. Dyn..

[133] Izrailev S., Stepaniants S., Balsera M., Oono Y., Schulten K. (1997). Molecular dynamics study of unbinding of the avidin-biotin complex. Biophys. J..

[134] Perilla J.R., Schulten K. (2017). Physical properties of the HIV-1 capsid from all-atom molecular dynamics simulations. Nat. Commun..

[135] Zhao G., Perilla J.R., Yufenyuy E.L., Meng X., Chen B., Ning J., Ahn J., Gronenborn A.M., Schulten K., Aiken C. (2013). Mature HIV-1 capsid structure by cryo-electron microscopy and all-atom molecular dynamics. Nature.

[136] Watanabe G., Sato S., Iwadate M., Umeyama H., Hayakawa M., Murakami Y., Yoneda S. (2016). Molecular Dynamics Simulations to Determine the Structure and Dynamics of Hepatitis B Virus Capsid Bound to a Novel Anti-viral Drug. Chem. Pharm. Bull..

[137] Sadati M., Ramezani-Dakhel H., Bu W., Sevgen E., Liang Z., Erol C., Rahimi M., Qazvini N.T., Lin B., Abbott N.L. (2017). Molecular Structure of Canonical Liquid Crystal Interfaces. J. Am. Chem. Soc..

[138] Popov P., Lacks D.J., Jákli A., Mann E.K. (2014). Insertion of liquid crystal molecules into hydrocarbon monolayers. J. Chem. Phys..

[139] Liu Q.Y., Zuo F., Zhao Z.-G., Chen J., Xu D. (2017). Molecular Dynamics Investigations of An Indicator Displacement Assay Mechanism in Liquid Crystal Sensor. Phys. Chem. Chem. Phys..

[140] Nguyen T.D., Carrillo J.-M.Y., Matheson M.A., Brown W.M. (2014). Rupture mechanism of liquid crystal thin films realized by large-scale molecular simulations. Nanoscale.

[141] Pollack L. (2013). Bionanotechnology and the Computational Microscope. http://www.ks.uiuc.edu/History/BioNano/.

[142] Popov P., Steinkerchner L., Mann E.K. (2015). Molecular dynamics study of rhodamine 6G diffusion at n-decane-water interfaces. Phys. Rev. E.

[143] Harder E., Damm W., Maple J., Wu C., Reboul M., Xiang J.Y., Wang L., Lupyan D., Dahlgren M.K., Knight J.L. (2016). OPLS3: A Force Field Providing Broad Coverage of Drug-like Small Molecules and Proteins. J. Chem. Theory Comput..

[144] Cipcigan F.S., Sokhan V.P., Crain J., Martyna G.J. (2016). Electronic coarse graining enhances the predictive power of molecular simulation allowing challenges in water physics to be addressed. J. Comput. Phys..

[145] Bi X., Hartono D., Yang K.-L. (2009). Real-Time Liquid Crystal pH Sensor for Monitoring Enzymatic Activities of Penicillinase. Adv. Funct. Mater..

[146] Hussain Z., Zafiu C., Küpcü S., Pivetta L., Hollfelder N., Masutani A., Kilickiran P., Sinner E.-K. (2014). Liquid crystal based sensors monitoring lipase activity: A new rapid and sensitive method for cytotoxicity assays. Biosens. Bioelectron..

[147] Van Duin A.C.T., Dasgupta S., Lorant F., Goddard W.A. (2001). ReaxFF: A Reactive Force Field for Hydrocarbons. J. Phys. Chem. A.

[148] Hu S., Sun W., Fu J., Zhang L., Fan Q., Zhang Z., Wu W., Tang Y. (2017). Reactive molecular dynamics simulations on the thermal decomposition of poly alpha-methyl styrene. J. Mol. Model..

[149] Delhommelle J. (2015). Recent advances in the molecular simulation of chemical reactions. Mol. Simul..

[150] Ribeiro J.V., Bernardi R.C., Rudack T., Stone J.E., Phillips J.C., Freddolino P.L., Schulten K. (2016). QwikMD—Integrative Molecular Dynamics Toolkit for Novices and Experts. Sci. Rep..

[151] Sheikh N.J., Sheikh O. Forecasting of biosensor technologies for emerging point of care and medical IoT applications using bibliometrics and patent analysis. Proceedings of the IEEE 2016 Portland International Conference on Management of Engineering and Technology.

[152] ProXentia Srl. http://www.proxentia.com/.

[153] Dynamic Biosensors Inc.. http://www.dynamic-biosensors.com/.

[154] Top Biosensor Companies. https://www.ventureradar.com/keyword/Biosensors.

[155] De Gennes P.G., Prost J. (1995). The Physics of Liquid Crystals.

[156] Xu M., Jones O.D., Wang L., Zhou X., Davis H.G., Bryant J.L., Ma J., Isaacs W.B., Xu X. (2017). Characterization of tubular liquid crystal structure in embryonic stem cell derived embryoid bodies. Cell Biosci..

